# Ultrasound contrast microbubbles to predict the microsphere distribution during transarterial radioembolization with holmium microspheres, an in vitro proof of concept study

**DOI:** 10.1080/10717544.2025.2505007

**Published:** 2025-05-18

**Authors:** Jan L. van der Hoek, Tess J. Snoeijink, Hadi Mirgolbabaee, Romaine Kunst, Michel Versluis, Jutta Arens, Srirang Manohar, Erik Groot Jebbink

**Affiliations:** ^a^ Multi-Modality Medical Imaging Group, TechMed Center, University of Twente, Enschede, The Netherlands; ^b^ Department of Medical Imaging, Radboud University Medical Center, Nijmegen, The Netherlands; ^c^ Physics of Fluids Group, TechMed Center, University of Twente, Enschede, The Netherlands; ^d^ Engineering Organ Support Technologies Group, Department of Biomechanical Engineering, University of Twente, Enschede, The Netherlands

**Keywords:** In-vitro model, transarterial radioembolization, ultrasound contrast microbubbles, holmium microspheres, biodistribution, particle flow behavior

## Abstract

Transarterial radioembolization (TARE) is an established treatment method for non-resectable liver tumors. One of the challenges of the approach is the accurate prediction of the microsphere biodistribution in the liver. We propose to use ultrasound contrast microbubbles as holmium microsphere precursors, which allows real-time prediction of the microsphere trajectories and biodistribution using dynamic contrast-enhanced ultrasound (DCE-US). The immediate goal in this in vitro study was to investigate the predictive capabilities of microbubbles as microsphere precursors. The study was conducted in an experimental in vitro model which represents the bifurcating right branch of the hepatic artery. A controlled injection of experimental BR-14 ultrasound contrast microbubbles and non-radioactive holmium-165 microspheres was performed in separate consecutive experiments in an arterial flow phantom. The microbubbles and microspheres were collected separately at the outlets of the phantom and counted using a Coulter counter to determine their distribution over the different outlets. The flow profile, the injection velocity, and the catheter position were monitored during the measurements to ensure stability. The results showed a good correlation between the microbubble and the microsphere distributions (p = 0.0038, r = 0.88) measured at the outlets. Differences in the distributions could be attributed to the characteristics of microbubbles and microspheres alone (e.g. particle size and concentration), since critical parameters were kept stable between the two experiments. The current in vitro study provides confidence that the microsphere biodistribution can be predicted using contrast microbubbles. The comparison provided by this study forms a foundation for the development of a DCE-US guided TARE treatment.

## Introduction

Primary liver cancer is the third leading cause of cancer-related mortality worldwide (Bray et al. 2018). The predominant type of primary liver cancer is hepatocellular carcinoma (HCC), which is resectable in only 20% to 30% of all cases (She and Chok 2015). Moreover, the liver is a common site for metastases originating from e.g. primary colorectal, pancreatic, and breast cancer (Horn et al. 2020), and these are inherently unresectable. For patients with well-defined but unresectable HCC nodules for intermediate stage HCC, locoregional therapies such as transarterial chemoembolization (TACE) and transarterial radioembolization (TARE) are the preferred treatment options (Salem et al. 2021; Reig et al. 2022). Although higher quality scientific evidence is available for TACE due to its earlier adoption, TARE has been suggested as an alternative to TACE due to its enhanced quality of life and reduced embolic volume, which helps preserve tumor feeding artery patency (Salem et al. 2013; Kloeckner et al. 2014; Brown et al. 2023). A recent patient data meta-analysis reported comparable overall survival for both treatments, with TARE offering an extended tumor progression time with generally less adverse effects (Brown et al. 2023).

In the TARE procedure, radioactive microspheres are injected via a microcatheter into the hepatic artery or one of its branches to target one or more liver tumors. Microspheres travel with the blood flow and lodge in the arterioles, interrupting the blood supply to the tumor and irradiating the tumors with radioactivity over time. In the traditional yttrium-90 (^90^Y) microsphere-based TARE procedure, the microsphere biodistribution can be determined post-TARE employing positron emission tomography (PET) or bremsstrahlung single-photon emission computed tomography (bSPECT) imaging. Alternatively, TARE can also be performed using holmium-166 (^166^Ho) microspheres (Quirem Medical B.V., Deventer, the Netherlands), which have a half-life of 26.8 hours compared to 64.1 hours for ^90^Y (Smits et al. 2010; Reinders et al. 2019).^166^Ho microspheres can also be analyzed by magnetic resonance imaging (MRI) and single-photon emission computed tomography/computed tomography (SPECT/CT), due to their paramagnetic properties and gamma emission, respectively (Smits et al. 2010; Klaassen et al. 2019). The medical imaging capabilities of ^166^Ho TARE have shown improved quantification and image quality of the biodistribution (van de Maat et al. 2013; Reinders et al. 2019).

Direct visualization of ^166^Ho microspheres during TARE is not possible with the currently employed imaging modalities, which poses a challenge for achieving a suitable biodistribution (Smits et al. 2012). Currently, a pretreatment scout procedure is used to predict the biodistribution of the radioactive microspheres, which takes place one or two weeks prior to the TARE procedure. In this scout procedure, a scout dose is injected that either contains Technetium-99 macroaggregated albumin (^99m^Tc-MAA) particles or a small amount of ^166^Ho microspheres (Smits et al. 2020). The scout procedure consists of one or multiple injection locations to target the liver tumors and this is replicated in the TARE treatment, if the biodistribution is considered sufficient in targeting the tumors. The scout procedure, however, has several limitations that reduce its predictive capabilities (Wondergem et al. 2013). First, the current scout procedure, comprising one or more catheter configurations (injection location from bifurcation, catheter tip radial position, injection velocity, etc.), can only predict one possible biodistribution at a time. Second, exact replication of the chosen catheter configuration in the scout procedure is difficult to achieve in the TARE treatment. While a close replication is achievable, a small shift in the catheter tip position from the optimal location by only 5 mm can already result in a significant deviation in the biodistribution by up to 40%, as shown by computational modeling (Basciano et al. 2010; Aramburu et al. 2017). Third, the commonly used particles for the scout procedure,^99m^Tc-MAA, have a size distribution (∼90% between 10 and 100 µm with a mean diameter of 35 µm) (Myers 2018; Jensen et al. 2022) that exceeds both lower and higher ends of the size distributions of ^90^Y and ^166^Ho microspheres, thereby possibly limiting their predictive accuracy (Wondergem et al. 2013). Last, the feedback from the scout procedure is not real-time, and a difference in the local hemodynamics during the scout procedure and that of the TARE treatment cannot be ruled out. The differences in injection configuration and hemodynamics can result in an off-target microsphere deposition, which leads to both increased off-target toxicity and decreased irradiation of liver tumors. The latter relates directly to the efficacy of the TARE procedure, as a sub-analysis of the SARAH trial showed better overall survival with a higher tumor radiation-absorbed dose (Hermann et al. 2020).

Real-time prediction of the ^166^Ho microsphere trajectories could improve the biodistribution, resulting in an improved treatment efficacy with less off-target toxicity. We hypothesize that real-time prediction could be realized by using ultrasound contrast microbubbles as microsphere surrogate particles. Ultrasound contrast microbubbles have an established diagnostic and therapeutic value in TARE (Lacerda et al. 2021). For instance, contrast microbubbles can be used to monitor the radiation response by visualizing changes in the microvasculature after TARE (Korpela and Liu 2014; Delaney et al. 2021). Another example of ultrasound microbubbles in TARE is the radiosensitization of tumors by local ultrasound-triggered microbubble destruction, which increases the treatment efficacy (Daecher et al. 2017; Eisenbrey et al. 2021). In addition to these applications, contrast microbubbles could enable prediction of the microsphere biodistribution, where dynamic contrast-enhanced ultrasound (DCE-US) could be used to visualize and quantify the presence of microbubbles (Lee et al. 2017). Contrary to the scout injection particles, microbubbles can pass through the complete vascular tree, so successive microbubble injections can be evaluated independently, without affecting the liver. Quantitative and real-time analysis of successive microbubble injections with DCE-US enables comparison of multiple catheter configurations (e.g. different catheter positions and injection velocities). As a result, the catheter configuration can be optimized when targeting a tumor before injecting microspheres.

However, the predictive capability of the microbubbles is yet to be established. Several in vivo studies in the closely related field of transarterial chemoembolization have investigated the injection of microbubbles to identify tumor feeding arteries (Moschouris et al. 2011; Burgmans et al. 2014; Wessner et al. 2020). In these studies, differences between the microbubble and chemoembolization particle behavior in the flow were not investigated, and the predictive capabilities of the microbubbles were limited to whether they ‘hit’ or ‘missed’ a tumor feeding artery. For the proposed DCE-US guided TARE approach, the behavior of microbubbles and ^166^Ho microspheres in the flow should be investigated. While ^166^Ho microspheres and microbubbles have different physical properties, it is yet to be determined whether these differences significantly influence their trajectories in the vasculature and result in different distributions.

This study aimed to explore the potential for a novel DCE-US guided TARE approach by comparison of the behavior of microbubbles and microspheres in the flow. We performed an in vitro study, using an experimental flow model representing the bifurcating right branch of the hepatic artery with physiologically relevant hemodynamics. The trajectories of ultrasound microbubbles and non-radioactive holmium-165 (^165^Ho) microspheres were both studied using a similar injection configuration. The number of microbubbles and microspheres that flowed to each outlet were determined using a Coulter counter (Multisizer 3, Beckmann Coulter Life Sciences, Woerden, The Netherlands), which served as a DCE-US surrogate for the microbubble quan­tification in vitro. The distributions were compared to establish the correlation in biodistribution of both entities. The immediate goal in this in vitro study was to investigate the predictive capabilities of microbubbles as microsphere precursors.

## Materials and methods

### Experimental model

**Figure 1.**
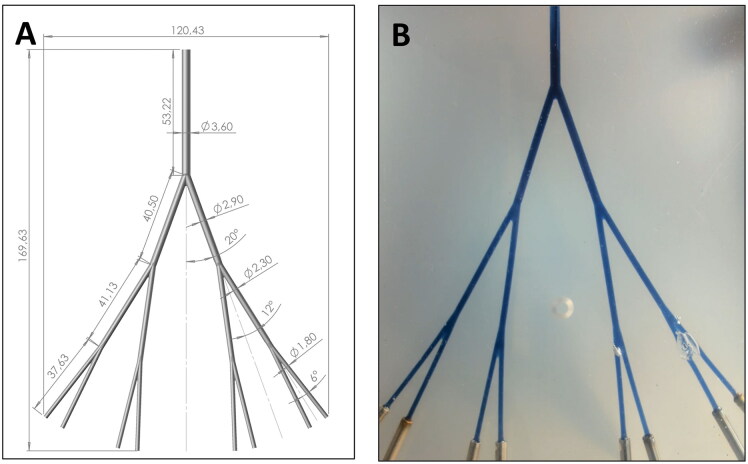
(A) The 3D CAD model representing a simplified right branch of the hepatic artery in SolidWorks (Dassault Systèmes, Wyman St Waltham, Massachusetts, USA) and (B) the RHA phantom made from PDMS including the capillary tubes, where the cavities represent the arterial lumen, visualized by filling the lumen with a mix of blue dye (Ecoline 533 indigo, Royal Talens, Apeldoorn, the Netherlands) and blood mimicking fluid.

A model representing the lumen of the right branch of the hepatic artery (RHA) was developed. The inlet diameter (3.6 mm) and angles of the model are based on the dimensions of the RHA lumen, as described by Basciano et al. (Basciano et al. 2010). The bifurcations in the model are symmetrical and the diameters of the branching vessels follow Murray’s law (Murray 1926). The resulting plane symmetric model has three bifurcation levels, resulting in eight outlets (Figure 1A).

The physical model was created by using a lost-core approach. First, the core model, representing the RHA vasculature, was made from acrylonitrile butadiene styrene (ABS) using a 3D printer (Ultimaker S5, Ultimaker, Utrecht, The Netherlands). Then, the RHA model was fixed in a Perspex container by clamping its inlet and outlets. Following this a polydimethylsiloxane (PDMS) Sylgard 184 (Dow Corning GmbH, Wiesbaden, Germany) solution was poured over the fixed ABS-printed RHA vasculature. The ABS was then dissolved by rinsing the model with acetone vapor, creating the physical rigid block phantom with cavities that represent the RHA lumen (Figure 1B). The final model was created by inserting capillary tubes in the outlets to ensure similar path lengths for all outlets. This model will be referred to as the RHA phantom.

### Experimental setup

**Figure 2.**
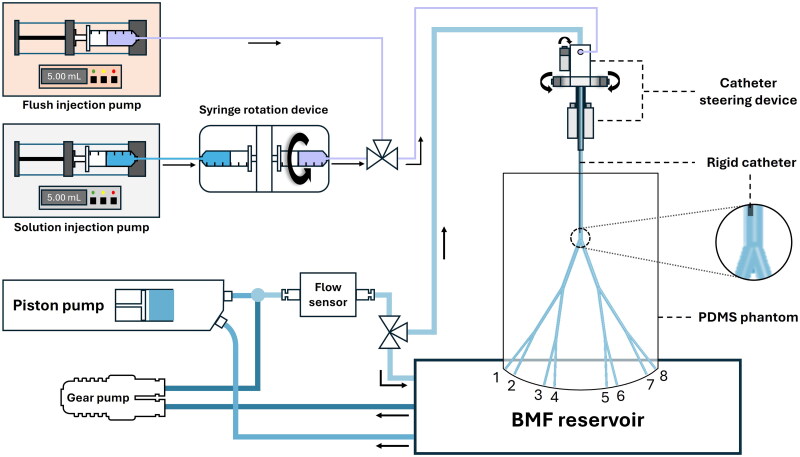
Schematic overview of the experimental in vitro setup used for the comparison between microbubble and microsphere trajectories.

The RHA phantom was installed in an open-flow circuit (Figure 2). The working fluid of the system is a Newtonian blood-mimicking fluid (BMF) solution, which was poured into a custom-made Perspex reservoir (BMF reservoir). The BMF solution consists of 55.8 wt% water, 34.2 wt% glycerol (99.7% purity, Laboratoriumdiscounter, IJmuiden, The Netherlands) and 10.0 wt% urea (99.3+% purity, Thermo Fisher Scientific Inc., Waltham, USA). The fractions were chosen to match both the density and viscosity of the solution with physiological values at room temperature, as well as to approximate the refractive index of PDMS (Brindise et al. 2018). The density of the fluid was measured with a density meter (DMA 35 Basic, Anton Paar GmbH, Graz, Austria), giving a density of 1111.8 kg/m^3^. The viscosity was measured with an Ubbelohde viscometer (SI Analytics GmbH, Mainz, Germany), resulting in a dynamic viscosity of 3.5 mPa·s.

The working fluid was pumped from the BMF reservoir to the other components in the setup with two pump types: a piston pump (SuperPump, ViVitro Labs, Victoria, Canada) to generate a pulsatile flow, and a gear pump (MGD1000P brushless micropump, RS Components Ltd., Corby, UK) to increase the mean flow rate. The outputs of both pumps were combined to create a waveform resembling flow in the RHA at 70 beats per minute (Aramburu et al. 2015). The waveform and flow rates were measured using a Coriolis flow sensor (MX55, Bronkhorst High-Tech B.V., The Netherlands), installed downstream of the pumps. The flow was then routed either directly to the BMF reservoir to tune the pump parameters for waveform replication, or toward the RHA phantom for measurements.

A custom-designed catheter steering device was installed upstream of the RHA phantom to enable precise positioning of the catheter for the injection of microbubbles and microspheres. The device, described in detail by Snoeijink et al. (Snoeijink et al. 2025), was used to steer the catheter in both the axial as well as the radial direction. A rigid capillary tube, further referred to as a rigid catheter, was mounted in the catheter steering device to minimize the movement of the catheter during injection. The dimensions of the rigid catheter (outer diameter = 0.9 mm, inner diameter = 0.6 mm) have been chosen to replicate the dimensions of a 2.7 F Progreat microcatheter (Terumo Interventional Systems, Zaventem, Belgium), which is a commonly used microcatheter type in TARE (Smits et al. 2020).

The solution injection pump (PHD ULTRA, Harvard Apparatus, Holliston, USA) was used to ensure precise and controlled delivery of the microbubble and microsphere solutions. This pump was hydraulically coupled to a syringe rotation device (Quirem Medical B.V., Deventer, The Netherlands), designed to maintain the injection solution in suspension. This device was validated for the administration of microspheres in a recent clinical study (van Wijk et al. 2024). In addition to the solution injection pump, the flush injection pump (NE-4000 Multi-Phaser^TM^, New Era Pump Systems, Farmingdale, USA) was installed to facilitate flushing of both the catheter and the injection line between the syringe and catheter.

The RHA phantom was placed above the BMF reservoir, allowing uncollected outflow to return directly to the reservoir. During measurements, fluid samples were collected in 50 mL centrifugal tubes, which were placed in custom-made aluminum sockets positioned to hold the tubes under each of the eight outlets.

### Injection characteristics

The microbubbles and ^165^Ho microspheres were injected in separate consecutive experiments, each while suspended within the syringe rotation device. BR-14 (Bracco Suisse SA, Geneva, Switzerland) polydisperse and experimental grade microbubbles were used with a mean measured diameter of 2.96 ± 3.98 µm, with the variation denoted by two times the standard deviation (±2σ), which has a right-skewed size distribution. BR-14 microbubbles have a similar size distri­bution as the clinically available Sonovue microbubbles (Sennoga et al. 2012), but with enhanced chemical and physical stability due to difference in shell composition (1,2-distearoyl-sn-glycero-3-phosphocholine (DSPC) and 1,2-dipalmitoyl-sn-glycero-3-phospho-(10-rac-glycerol) (DPPG)) and core composition (perfluorobutane (C_4_F_10_) and dinitrogen (N_2_)). This enhanced stability was required for prolonging the time available for analysis. The injection solution for each microbubble injection was created by resuspending one BR-14 vial with 5 mL of phosphate buffered saline (PBS) solution (Dulbecco’s phosphate buffered saline, Sigma-Aldrich, St. Louis, USA). Non-radioactive particles containing holmium-165 in a poly-L-lactic acid (PLLA) shell (Quirem Medical B.V., Deventer, The Netherlands) were used for the microsphere injections. These ^165^Ho PLLA microspheres had a measured diameter of 28.94 ± 7.07 µm, with a reported density of 1.41 g/mL (QuiremSpheres^TM^ Microspheres 2024). In each microsphere measurement, a solution was injected that contained 300 mg of ^165^Ho microspheres, 4.5 mL PBS, and 0.5 mL of a pluronic buffer solution solvent (0.1% pluronic, Quirem Medical B.V., Deventer, The Netherlands), where the latter facilitated the homogeneous suspension of the microspheres (Zielhuis et al. 2005).

The injection of the microbubble or microsphere suspension was initiated approximately 10 seconds after starting the flow through the RHA phantom. Using the solution injection pump, the suspension was injected at a continuous flow rate of 10 mL/min, resulting in an injection duration of 30 seconds. Directly afterwards, the flush injection pump used the same configuration to flush the injection line and the catheter with 5 mL of PBS. After completing the flush, flow through the RHA phantom was extended for another 10 seconds to direct any residual particles left in the RHA phantom to the outlets.

### Catheter position analysis

**Figure 3.**
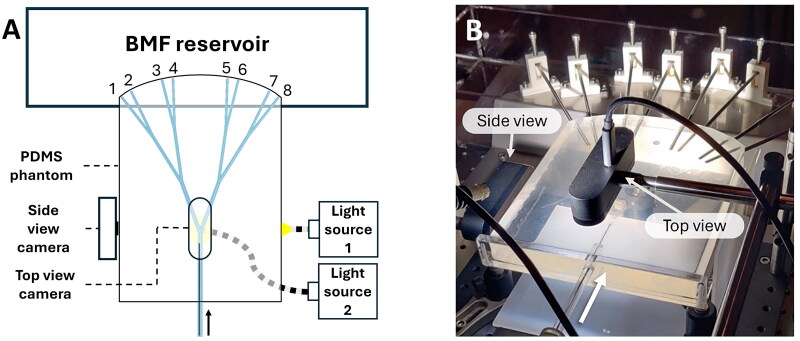
(A) A schematic overview and (B) a photo of the right branch of the hepatic artery phantom, including the cameras and LED light sources for analysis of the catheter position and movement. Note that Figure 3A is flipped with respect to Figure 2.

**Figure 4.**
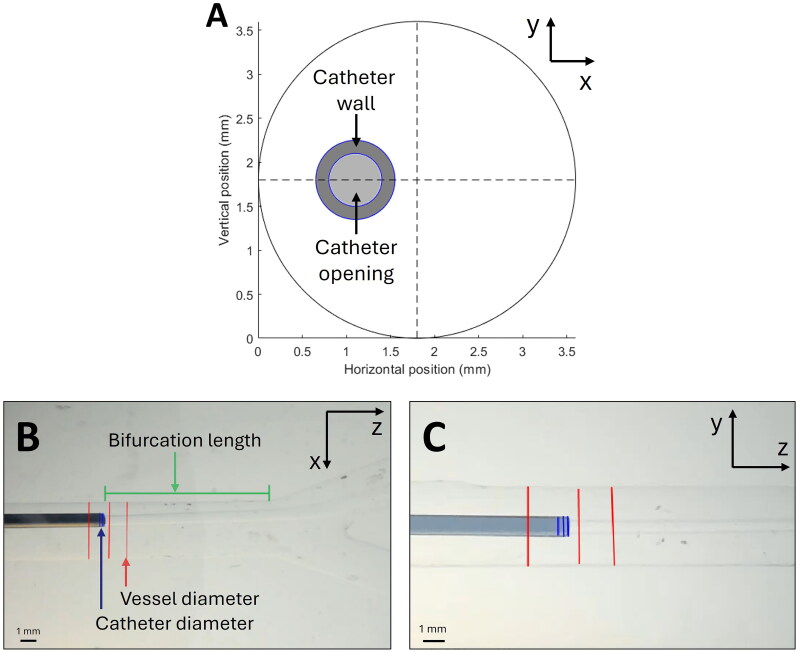
Analysis of the catheter position, with (A) the desired 9 o’clock position of the catheter tip, showing the catheter opening (light grey) and catheter wall (dark grey) in the cross-section of the right branch of the hepatic artery phantom, and (B, C) the measurements of the catheter tip location on the first frame for both the top view (B) as well as the side view (C). Reference lines were used for measuring the catheter tip location, where the blue vertical lines indicate the diameter of the catheter, the red lines indicate the lumen diameter, and the green line (B) indicates the distance between the catheter and the start of the bifurcation fixed at a distance of 11.1 mm.

To determine and monitor the position of the catheter during measurements, two cameras (Logitech BRIO, Logitech, Lausanne, Switzerland) were installed above and next to the RHA phantom to create top and side view videos (Figure 3 and Figure 4). The contrast-to-background of the catheter was increased by installing two LED light sources (KL 2500 LED, Schott AG, Delligsen, Germany) below and next to the transparent RHA phantom, to back-illuminate the catheter.

The same catheter position was used in all measurements. The catheter was positioned to target the left side of the RHA phantom (Figure 4A), referred to as the 9 o’clock position (*x* = 1.1 mm and *y* = 1.8 mm). The axial position of the catheter tip was placed at 11.1 mm from the start of the bifurcation (Figure 4B). The catheter was aligned coaxially with the vessel wall to minimize the angle of injection. A Matlab script (MATLAB R2022b; MathWorks Inc, Natick, Massachusetts) was used to create a live overlay of the desired position on both camera views for the initial positioning of the catheter and confirming this position before each measurement.

The position and movement of the catheter tip were determined by capturing the injection on both cameras. Using the first frame of both views, the catheter diameter, as well as the RHA phantom entrance diameter, were measured three times along the length (red and blue lines in Figure 4B and Figure 4C). These measurements were averaged to determine the initial catheter position. The movement of the catheter tip during injection was determined by segmentation by an RGB-based color threshold method (Chong et al. 2020). The catheter tip was segmented in each frame for both views, and the centroid of each segmented catheter tip was calculated. The movement of the catheter tip was obtained by calculating the centroid displacement per frame. Using the initial tip position of the catheter, the position during the complete injection was determined for both the top and side view and combined to give the catheter position in the injection plane over time. The result was presented in a heat map, which represents the catheter tip location in the injection plane, expressed as a fraction of its presence at a specific location with respect to the entire injection duration.

### Sample analysis

The distributions of microbubbles and microspheres over the outlets were analyzed from collected fluid samples at all eight outlets, repeated five times for both types for averaging and repeatability. Before starting the measurements, samples were collected and weighed in a calibration measurement to ensure equal volumetric outflow at each outlet of the RHA phantom.

The number of particles in the fluid-filled tubes was determined using a Coulter counter (Multisizer 3, Beckmann Coulter Life Sciences, Woerden, The Netherlands). The particle diameter range that can be counted accurately is determined by the used aperture tube, which has a lower limit of 2% and an upper limit of 60% of its nominal diameter size. Here, the microspheres were counted using a 100-µm aperture tube (covering the particle diameter range of 2.0 µm − 60 µm), thus measuring the full-size range of the microspheres. The microbubbles were counted using the 70 µm aperture tube (1.4 µm − 42 µm), which accounts for the majority of the microbubble size range while excluding submicron contaminants that may have mixed with the working fluid (BMF).

To determine the number of particles per tube, a 1 mL sample of each tube was measured using the Coulter counter, as follows: first, all tubes were weighed to account for volume differences between the tubes. Then, the microbubbles and microspheres in the tubes were homogeneously suspended for accurate sampling. The tubes that contained microbubbles were gently swirled (1 revolution per second at an angle of 45°, approximately) to create a homogeneous suspension without destroying the microbubbles. The tubes that contained microspheres were suspended using a vortex mixer (Corning LSE Vortex Mixer, Corning Inc., New York, USA). After mixing the particles, 1 mL of each sample was pipetted into 50 mL of Isoton II diluent (Beckmann Coulter Life Sciences, Woerden, The Netherlands). Lastly, the number of particles in the diluted solution was determined by taking three measurements from the solution using the Coulter counter and averaging the results.

The output of the Coulter counter categorizes the number of particles per bin size. First, the cumulative sum over all bins was calculated to obtain the number of particles in each sample. Delimiters were employed to filter out noise and account for the aperture’s bandwidth. For the microbubbles, the aperture limits were used as outer bounds (1.4 µm − 42 µm). For the microspheres, the limits for the sum were set around the mean microsphere diameter at 20 µm and 40 µm. Then, the total amount of particles in each tube was obtained by multiplication with the dilation factor and the weight of each tube. Lastly, a correction was applied to the total amount of particles to account for the number of background particles in BMF, determined by measuring a BMF sample using the Coulter counter. Particle numbers were then compared for each measurement and normalized to obtain the percentual distribution.

The microbubble and microsphere distributions were compared using the mean values, standard deviations (±2σ) and the Pearson’s correlation coefficient (*r*, *p*).

## Results

### Waveform

**Figure 5.**
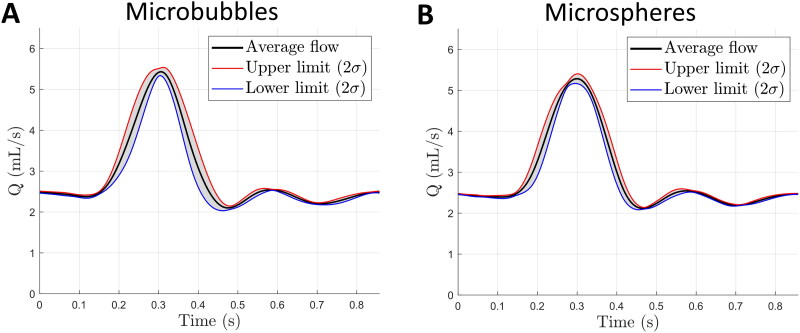
Mean waveforms and standard deviations of (A) the BR-14 microbubble measurements and (B) the ^165^Ho microsphere measurements.

The mean flow waveform over all BR-14 microbubbles and all ^165^Ho microsphere measurements was calculated (Figure 5). The mean waveforms were calculated from the values of the average waveforms, which consist of 60 subsequent beats for each measurement. The values for the microbubble and microsphere mean average waveforms are mean flow (*Q* = 2.90 ± 0.02 mL/s, vs. *Q* = 2.89 ± 0.01 mL/s), peak systolic flow (*Q* = 5.66 ± 0.10 mL/s, vs. *Q* = 5.53 ± 0.12 mL/s), and peak diastolic flow (*Q* = 1.99 ± 0.06 mL/s vs. *Q* = 1.98 ± 0.04 mL/s). The waveforms and standard deviations of each measurement can be found in Supplemental Materials 1.

### Catheter location

**Figure 6.**
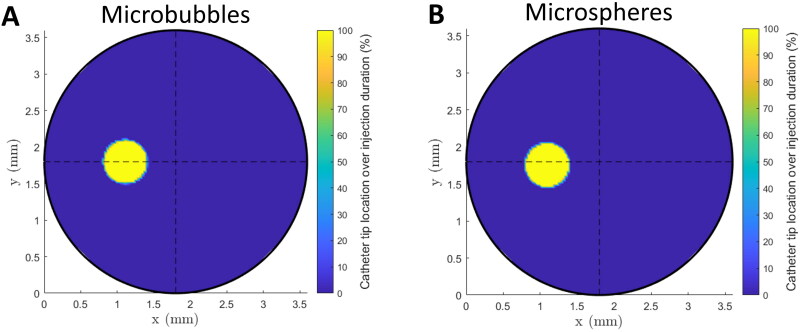
Heat maps of the average catheter location and movement of (A) the BR-14 microbubble measurements and (B) the ^165^Ho microsphere measurements. The position of the catheter tip is expressed as a percentage of its presence at a location over the total duration of the injection, in the injection plane.

The radial position of the catheter within the cross-section is provided in Figure 6. A movement of the catheter was found that corresponded to the pulsatility of the waveform. The resolution used for the plots corresponds to the largest pixel size found in all top view and side view videos (*Δ* = 0.033 mm), using the highest camera settings (3840 *×* 2160 pixels, framerate = 30 Hz). The catheter position and displacement over all microbubble and microsphere measurements are average horizontal position (*x* = 1.11 ± 0.02 mm vs. *x* = 1.10 ± 0.02 mm), average vertical position (*y* = 1.80 ± 0.02 mm vs. *y* = 1.76 ± 0.02 mm), average maximum horizontal displacement (*Δx* = 0.03 ± 0.01 mm vs. *Δx* = 0.02 ± <0.01 mm), and average maximum vertical displacement (*Δy* = 0.04 mm, *SD* = 0.04 mm vs. *Δy* = 0.02 ± <0.01 mm, *SD* = < 0.01 mm). The axial position of the catheter with respect to the start of the bifurcation was constant over all experiments, at *z* = 11.10 mm. Heat maps of all measurements can be found in Supplemental Materials 2.

### Microbubble and microsphere distributions

**Figure 7.**
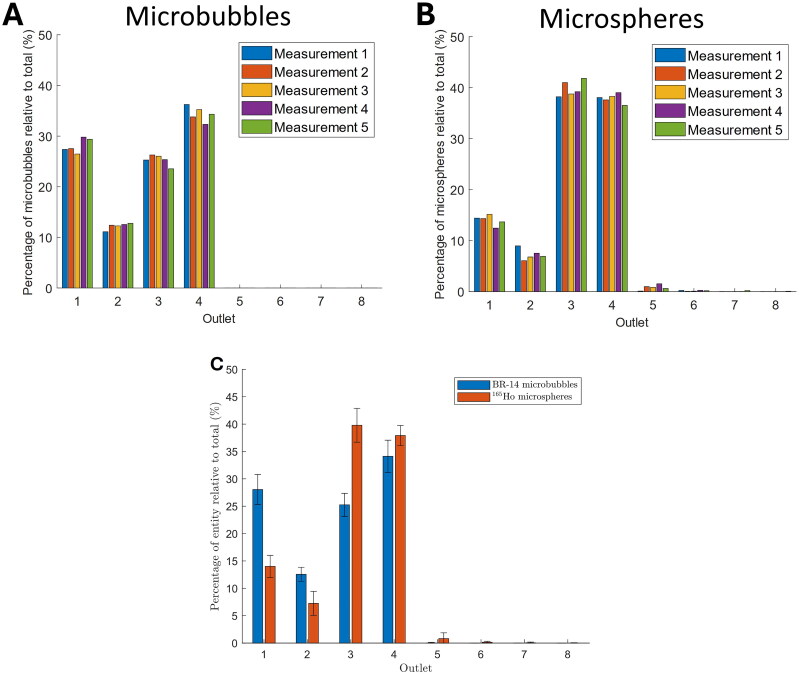
(A) BR-14 microbubble and (B) ^165^Ho microsphere distributions for all measurements, expressed relative to the total number of particles that have been measured over all outlets per measurement. (C) Average distributions of the microbubbles (MB) and microspheres (MS) including standard deviations (2σ), expressed as a percentage of the total number of particles in all outlets.

The distributions of the individual microbubble and microsphere measurements can be found in Figure 7A and Figure 7B. The average distributions and SD can be found in Figure 7C and Table 1. The particle counts, measured per outlet, are provided in Supplemental Materials 3. The correlation between both distributions, averaged over all outlets, is *p* = 0.0038, *r* = 0.88. Notable differences between the distributions over the different outlets were observed, with the largest percentual differences of approximately 14% found in outlets 1 and 3. The volumetric distribution from the initial experiment to measure outflow equality shows a maximum deviation of 0.8% over all eight outlets.

**Table 1.** Average relative distributions of the microbubbles and microspheres over the eight outlets, including standard deviations (SD, 2σ), corresponding to Figure 7C.

Outlet	Microbubbles % (SD)	Microspheres % (SD)
1	28.0 (2.8)	14.0 (2.0)
2	12.6 (1.3)	7.3 (2.2)
3	25.3 (2.1)	39.8 (3.1)
4	34.1 (2.9)	37.9 (1.8)
5	0.0 (0.1)	0.8 (1.0)
6	0.0 (0.1)	0.2 (0.2)
7	0.0 (0.0)	0.0 (0.1)
8	0.0 (0.0)	0.0 (0.0)

## Discussion

To investigate the potential of DCE-US guided TARE, the distributions of ultrasound contrast microbubbles and ^165^Ho microspheres were compared in an in vitro RHA phantom. Analysis of the distributions of both microparticles averaged over all outlets showed a moderately strong correlation (*p* = 0.0038, *r* = 0.88). Microbubbles and microspheres were found in similar outlets, but were only comparable in number for outlet 4 (Figure 7). No particles were found in outlets 5 to 8, which confirms the intended catheter placement to target only the left side of the phantom. Also, outlet 2 received the lowest fraction of particles for both microbubbles and microspheres in the outlets with a significant deposition (outlets 1 to 4). However, a more homogeneous distribution for microbubbles was found over the remaining targeted outlets (outlets 1, 3, and 4), whereas the microspheres distribution was more focused on the inner outlets of the targeted side (outlets 3 and 4). Percentual differences between the distributions of approximately 14% were found in outlets 1 and 3, indicating that outlet 1 received twice as many microbubbles as microspheres. The moderately strong correlation indicates that prediction of the microsphere deposition using DCE-US should be feasible.

The accuracy of the flow behavior comparison between microbubbles and microspheres in the RHA phantom largely depends on the parameter similarity between measurements. Several parameters and their influence on the microsphere distribution were discussed in the systematic review by Snoeijink et al., identifying the axial and radial catheter tip position and the injection velocity as the most impactful parameters (Snoeijink et al. 2023). The variations in the catheter tip position were limited by a rigid catheter, which was mounted in the injection device to prevent displacement in the axial direction and limit displacement in the radial direction caused by the flow pulsatility. As a result, a similar catheter tip location from the bifurcation was found in all experiments (z = 11.10 mm). For the radial catheter tip positions, slight deviations were found for the microbubble (x = 1.11 mm (30.8% with respect to the inlet diameter) and y = 1.80 mm (50.0%) and microsphere (x = 1.10 mm (30.6%) and y = 1.76 mm (48.9%) injections. A slight displacement of the catheter tip position can significantly change the particle trajectories (Aramburu et al. 2017; Taebi et al. 2022), and these deviations could have been attributed to the observed differences in the distributions over the outlets. The injection velocity was kept constant in all experiments by using a programmable syringe pump. The pump specifications indicate accuracy and reproducibility of ± 0.25% and ± 0.05% respectively (PHD ULTRA Programmable Syringe Pump - Darwin Microfluidics 2024), implying a negligible effect on the microbubble and microsphere distributions. Additional parameters have been identified in other studies that have a significant impact on the microsphere distribution, such as the inlet flow profile and magnitude expressed as a Reynolds number by (Amili et al. 2019). A comparison of the flow profiles between the microbubble and microsphere measurements shows comparable mean flow and peak diastolic flow. A slight deviation was found between the peak systolic flows. However, this deviation is much smaller than the difference analyzed in the study by Amili et al. and is thus expected to have a negligible effect on the found distribution.

The similarity of parameters in the experiments suggests that the differences in distribution over the outlets are primarily driven by the physical property variations between microbubbles and microspheres. The behavior of particles suspended in a flow can be expressed by the Stokes number, which indicates that the particle diameter and the density influence its trajectory. Indeed, the mean diameter of microbubbles and microspheres differs by an order of magnitude (∼3 µm vs. ∼30 µm), and the density differs due to different core compositions, namely gaseous for microbubbles, and solid for microspheres. The relatively larger Stokes number belonging to the microspheres implies a more dominant particle inertia, which is likely to be the cause of the microspheres’ slight tendency toward the inner outlets (3 and 4). However, the calculated Stokes number (St ≪ 0.1) suggests that both microbubbles and microspheres should follow the flow direction closely. The difference in distribution over the outlets could also result from differences in the amount of injected particles between microbubbles (∼1 − 2.5 billion per 5 mL (Safety Data Sheet SonoVue 2025)) and microspheres (∼17 million per 5 mL). This increase can lead to different particle-particle interactions in the flow, which impact the particle spread in the RHA phantom. Consequently, reducing the concentration of the injected microbubbles could improve the distribution similarity.

While differences are required in TARE, to ensure that only microspheres lodge in the microvasculature, optimizing the characteristics of both microbubbles and microspheres could enhance the predictive accuracy of DCE-US-guided TARE. For microbubbles, several commercialized options exist that differ in core gas composition, shell type, and mean diameter (Lee et al. 2017; Frinking et al. 2020). While the type of core gas and shell could increase the microbubble density, such differences will be small compared to the relatively high density of the microspheres. Concerning the mean diameter, other types of polydisperse microbubbles have been reported with slightly higher mean diameters, however, the impact on the microbubbles’ distribution is also expected to be negligible. Alternatively, monodisperse microbubbles could be used to increase the mean diameter while still allowing the microbubbles to pass through the capillaries. In addition to being more similar in size to microspheres, monodisperse microbubbles can provide sufficient ultrasound signal enhancement at relatively lower concentrations (Helbert et al. 2020), potentially altering their trajectories by reducing particle interactions. Currently, however, clinical approval of monodisperse microbubbles is still in progress (Frinking et al. 2020).

For microspheres, two other commercially available types exist besides ^166^Ho PLLA microspheres, which both contain the radioactive isotope ^90^Y (d’Abadie et al. 2021). While the ^90^Y-Resin microspheres (Sir-Spheres, Sirtex Medical Ltd., Sydney, Australia) have both a higher density and mean diameter, the ^90^Y-Glass microspheres (Therasphere, Boston Scientific, Boston, Massachusetts, USA) have a slightly lower mean diameter (25 µm vs. 30 µm). However, the density of these spheres is more than twice that of ^166^Ho PLLA microspheres, which could lead to larger distribution differences, while also compromising the diagnostic capabilities offered by the ^166^Ho PLLA microspheres. The current prediction used in TARE is the so-called pretreatment scout procedure. In addition to the prediction of the microsphere biodistribution, the scout procedure is also used for determining the radiation dose and establishing whether lung shunting, and extrahepatic deposition occur for the chosen catheter configuration. DCE-US prediction cannot determine the radiation dose, nor lung shunting, as the small size of the microbubbles allows them to pass through capillaries and reach the lungs. Some extrahepatic depositions could be predicted using the DCE-US approach, as the ultrasound probe can be relocated to scan organs surrounding the liver for microbubble presence where extrahepatic depositions are common, such as the gallbladder and duodenum, although the latter might be challenging due to its deep anatomical location (Etzel et al. 2024). The extrahepatic deposition is an absolute contraindication for TARE, where a different injection configuration (e.g. a more proximal injection location to avoid an extrahepatic collateral artery) could solve this problem, which is facilitated by the direct and real-time analysis of the microbubble injections (Weber et al. 2022). Replacement of the entire scout procedure by the DCE-US prediction is unlikely, and it is currently envisioned as an additional step in the treatment phase.

The DCE-US prediction can be incorporated into the TARE procedure at the start of the treatment. First, the catheter is placed at the initially selected position from the scout procedure. Then, an ultrasound probe is placed at the tumor location, and microbubbles are injected via the catheter instead of microspheres. The signal intensity of the microbubbles is measured at the target location using DCE-US, which results in a time-intensity curve. The suitability of the catheter position is assessed based on characteristics of this curve, such as the peak intensity. The catheter configuration is then refined through an iterative process, comparing multiple catheter configurations by their resulting time-intensity curves. The most suitable configuration is then selected, e.g. by choosing the configuration that yielded the highest peak intensity. Using this configuration, the ultrasound probe is relocated to predict depositions in healthy liver tissue, as well as extrahepatic depositions. If the final deposition is deemed suitable, radioactive microspheres are injected using the same configuration for an optimized microsphere distribution. Since microspheres lodge in the arterioles after injection, the DCE-US prediction of the microsphere biodistribution corresponds to the tissue that is irradiated over time.

For the prediction of the biodistribution of the microspheres, the DCE-US prediction has some clear advantages over the scout procedure prediction. First, the real-time prediction by DCE-US eliminates differences that arise from catheter configuration variations and hemodynamic differences inherent to the scout procedure approach (Chiesa and Maccauro 2020; Smits et al. 2020; Weber et al. 2022). The observed differences in the microbubble and microsphere distributions in this study are considered minor with respect to the accuracy of the scout procedure prediction. Second, a radioactive scout dose with a deposition outside of the target area results in damage to healthy tissue, whereas microbubbles spare healthy tissue in such cases with a low prevalence of minor adverse effects (1 in 3000 examinations) (Shang et al. 2023). Last, the injection configuration can be optimized using direct and quantitative feedback, unlike the current prediction, which requires significant time per iteration.

### Study limitations

A simplified version of the RHA with symmetrical bifurcations was chosen as the experimental model, such that only a small part of the vasculature that is relevant for TARE is represented. The current comparison holds for prediction of the distribution in different lobes or for the distribution in major parts of a liver lobe. The vasculature level where embolization takes place is not included in the RHA phantom, such that the prediction of the microsphere distribution might not hold for smaller liver lobe sections. Additionally, the RHA phantom was designed to have an equal outflow in all outlets to highlight the distribution differences between microbubbles and microspheres over the outlets. In a cancerous liver, local hemodynamics can be altered by phenomena such as intratumoral arteriovenous shunts and angiogenesis. The current experimental model lacks these features and therefore does not represent a cancerous liver.

The experiment was designed with a focus on parameter stability and reproducibility of the results. The comparison in this study is based on the analysis of one injection configuration that is feasible in TARE, limiting the analysis scope. Other injection configurations result in different distributions and the correlation might change for other configurations. However, other factors could also impact this correlation, such as a more complex vascular network and non-Newtonian blood flow. Therefore, the assessment of the correlation within this in vitro setup is limited.

## Conclusions

This study demonstrates the potential of ultrasound contrast microbubbles to predict the microsphere biodistribution in TARE. Analysis of the averaged microbubble and microsphere distributions in the RHA in an in vitro experimental model showed a moderately strong correlation (*p* = 0.0038, *r* = 0.88). Variations in other parameters that could influence the particle trajectories were negligible, such that the differences in distribution could be distilled down to differences in physical properties between microbubbles and microspheres. The correlation between microbubbles and microspheres shows promise for the prediction of the microsphere biodistribution, laying the foundation and establishing the potential for DCE-US guided TARE. Although a good correlation was observed, the microspheres exhibited a slightly more focused distribution compared to a more homogeneous distribution for the microbubbles.

Clinical implementation of the DCE-US guided TARE approach requires development of this method in a more relevant setting. Future research should focus on the development of the ultrasound techniques for determining the microbubble distribution in liver tissue. Also, other parameters such as the injection location and injection velocity could be explored in this setting, to further investigate the potential and limitations of the prediction of the microsphere biodistribution with DCE-US. Once the DCE-US guided TARE protocol has been established, clinical trials could explore the potential of this approach to improve patient outcomes.

## Supplementary Material

Supplemental Material

## Data Availability

The authors confirm that the data supporting the findings of this study are available within the article and its supplementary materials.
