# Supplementary figures and images for: Ultrasound contrast microbubbles to predict the microsphere distribution during transarterial radioembolization with holmium microspheres, an in vitro proof of concept study
Source: Drug Deliv. 2025 May 18;32(1):2505007. doi: 10.1080/10717544.2025.2505007 (PMC12090288; doi:10.1080/10717544.2025.2505007)

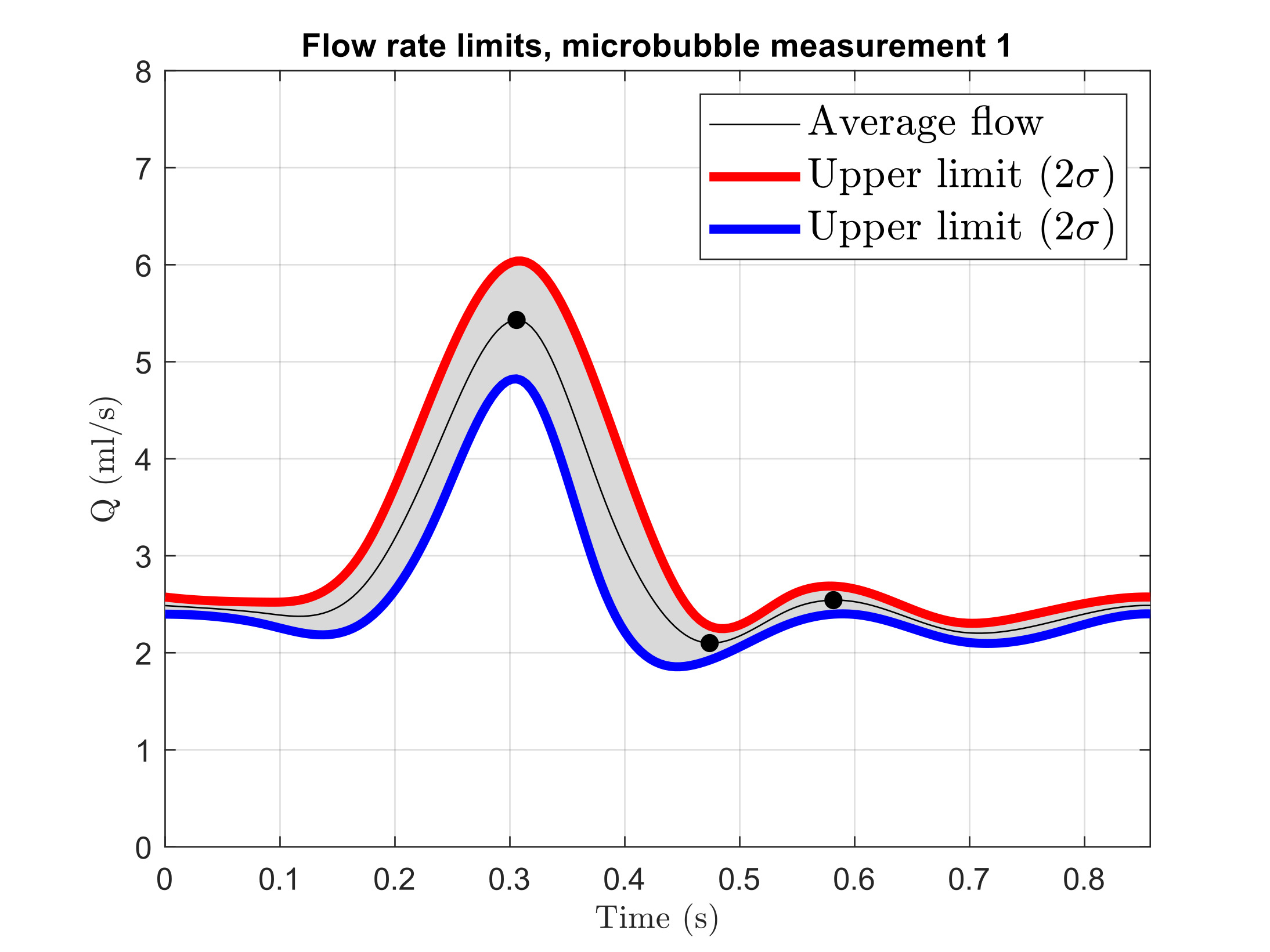

Supplement: Supplemental Material [file IDRD_A_2505007_SM5900.zip › Suppl_Doc/Sup1_Bubble_flow_1.jpg]

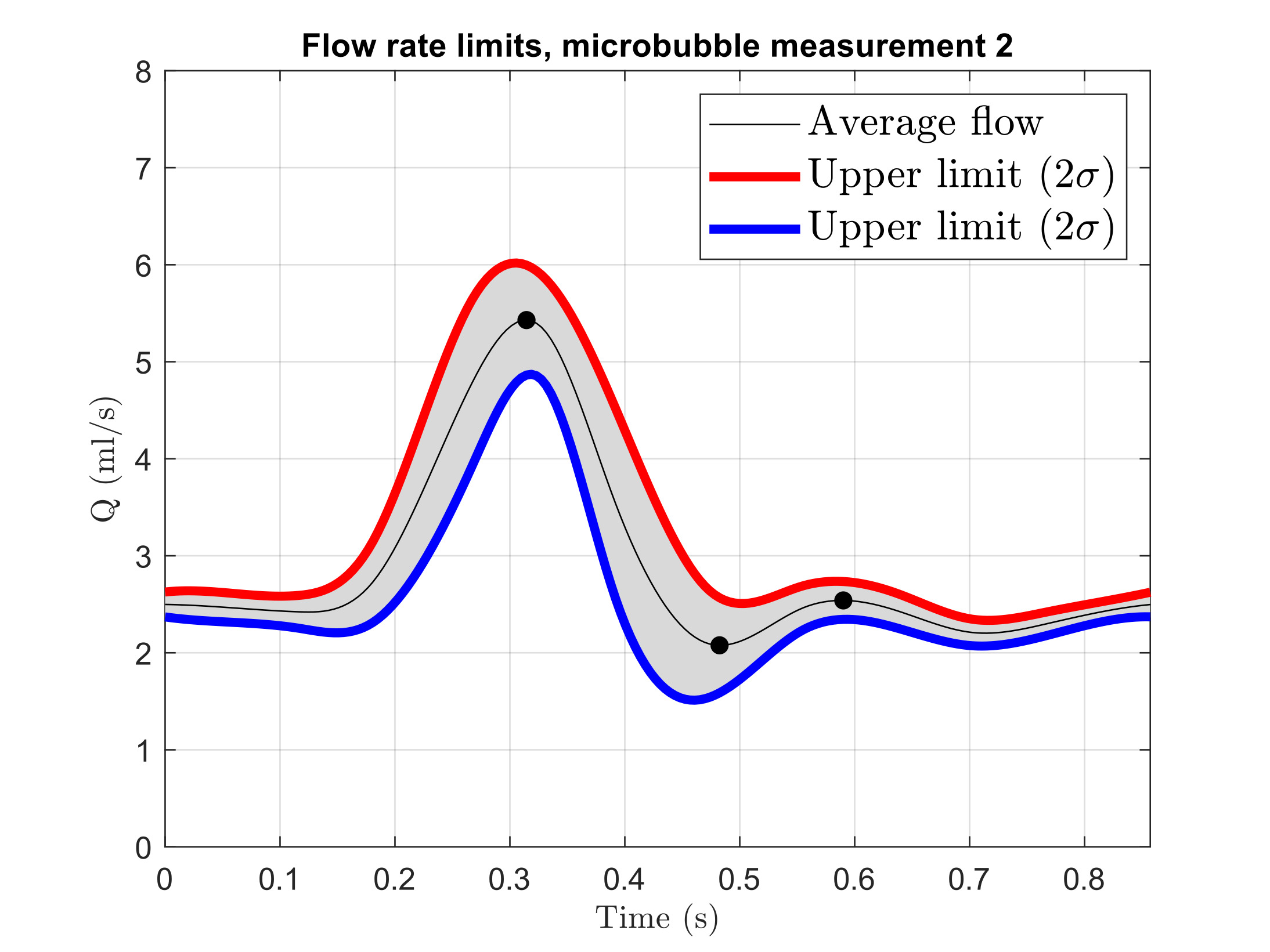

Supplement: Supplemental Material [file IDRD_A_2505007_SM5900.zip › Suppl_Doc/Sup1_Bubble_flow_2.jpg]

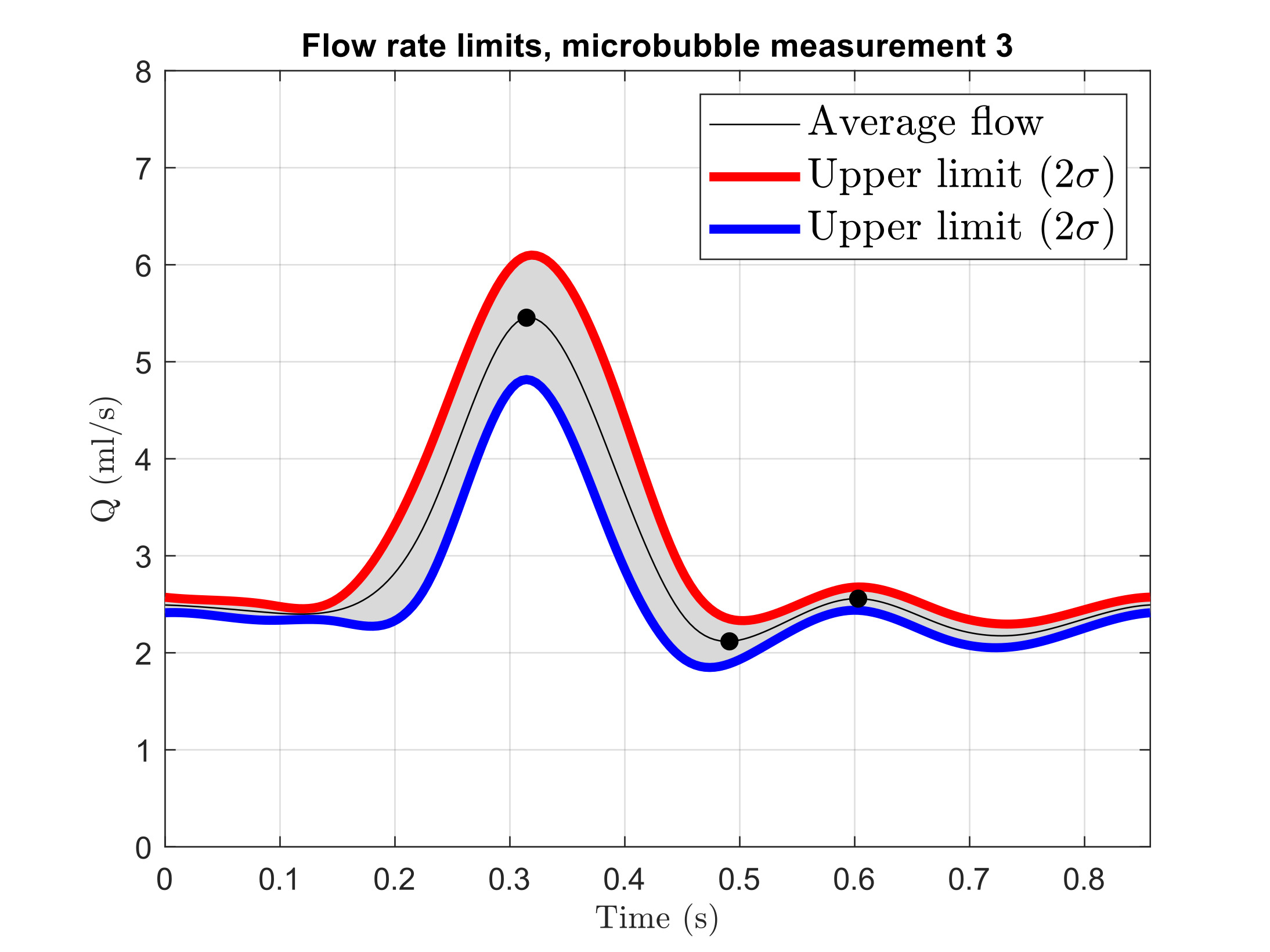

Supplement: Supplemental Material [file IDRD_A_2505007_SM5900.zip › Suppl_Doc/Sup1_Bubble_flow_3.jpg]

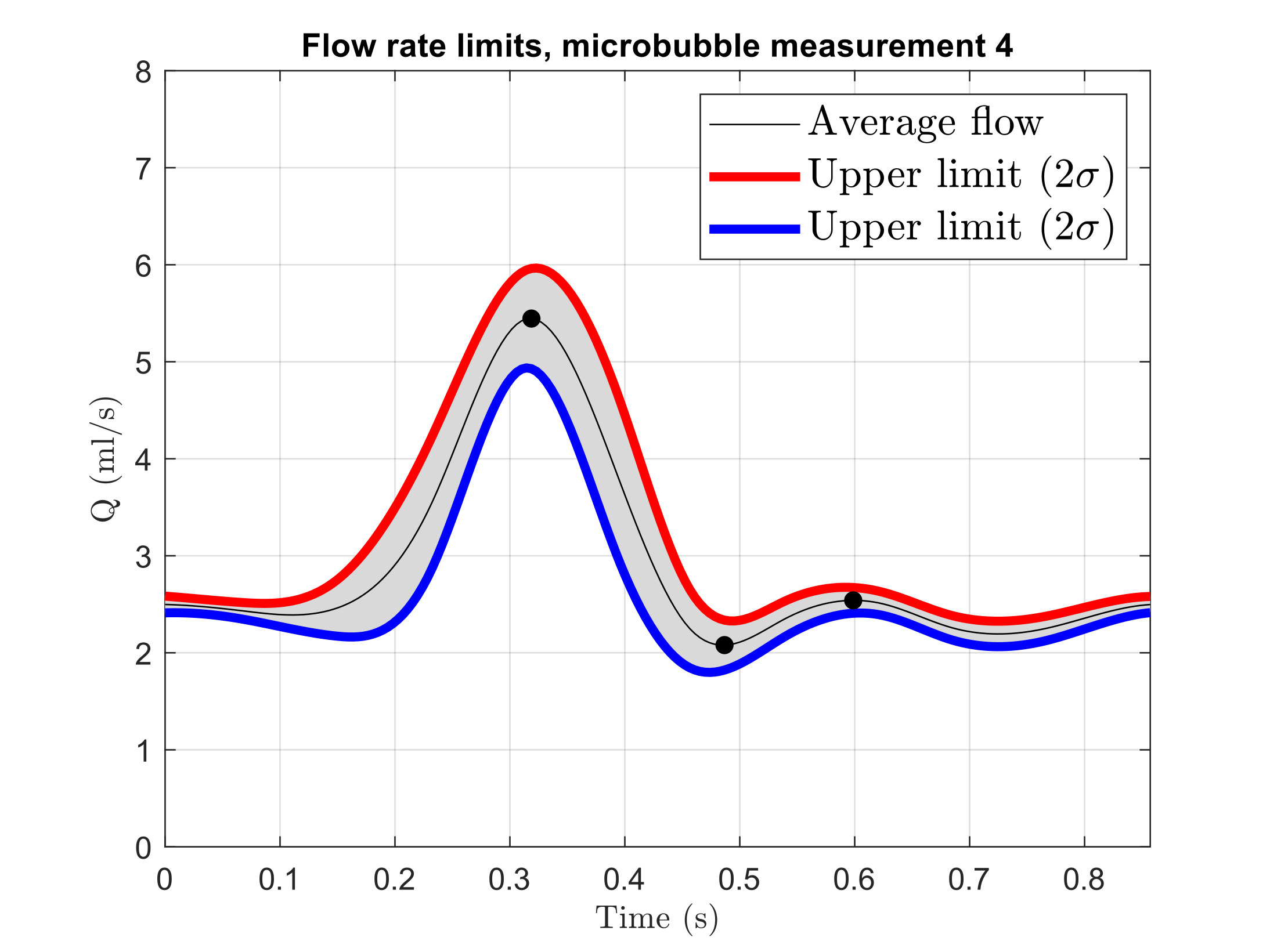

Supplement: Supplemental Material [file IDRD_A_2505007_SM5900.zip › Suppl_Doc/Sup1_Bubble_flow_4.jpg]

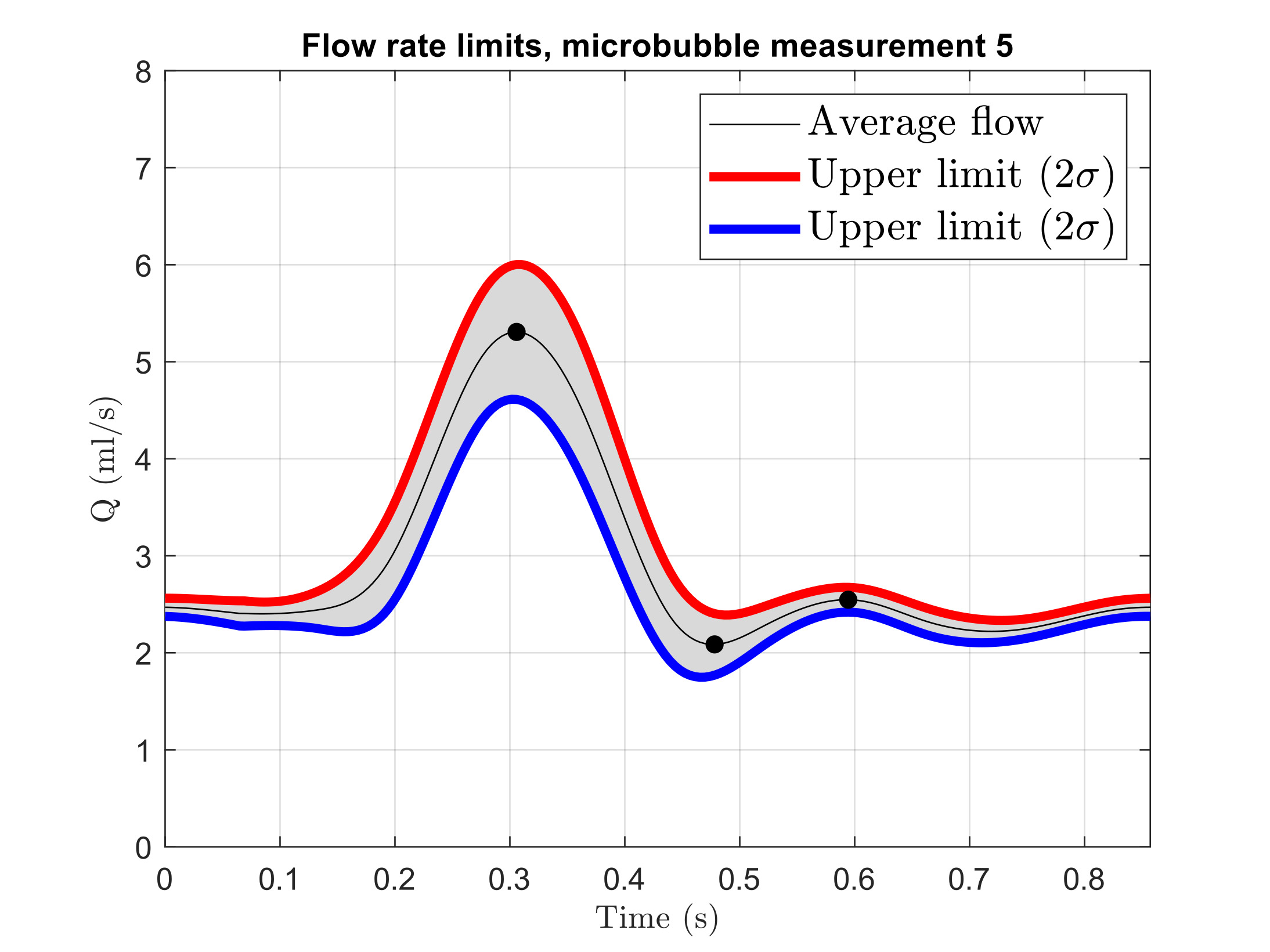

Supplement: Supplemental Material [file IDRD_A_2505007_SM5900.zip › Suppl_Doc/Sup1_Bubble_flow_5.jpg]

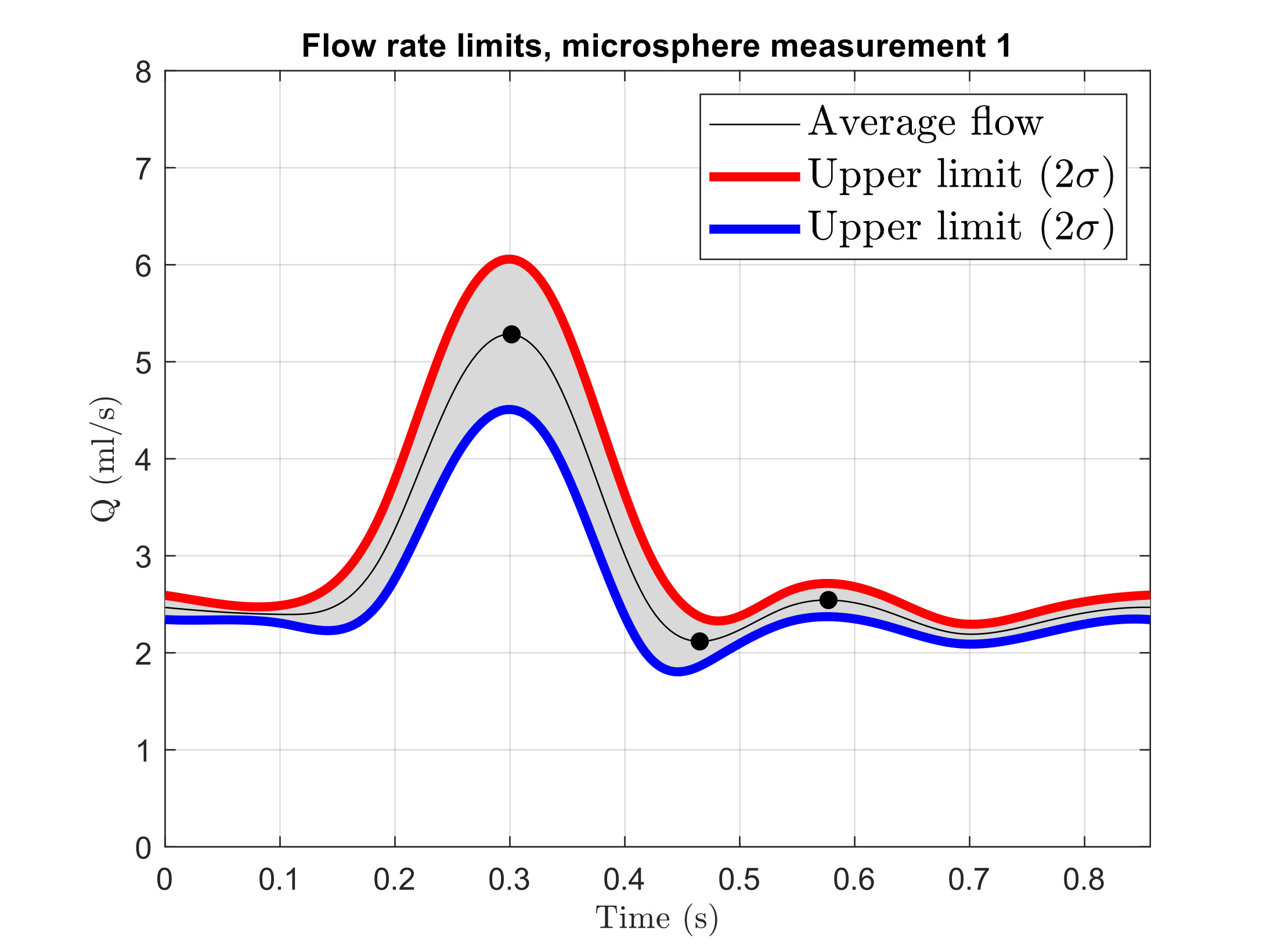

Supplement: Supplemental Material [file IDRD_A_2505007_SM5900.zip › Suppl_Doc/Sup1_Holmium_flow_1.jpg]

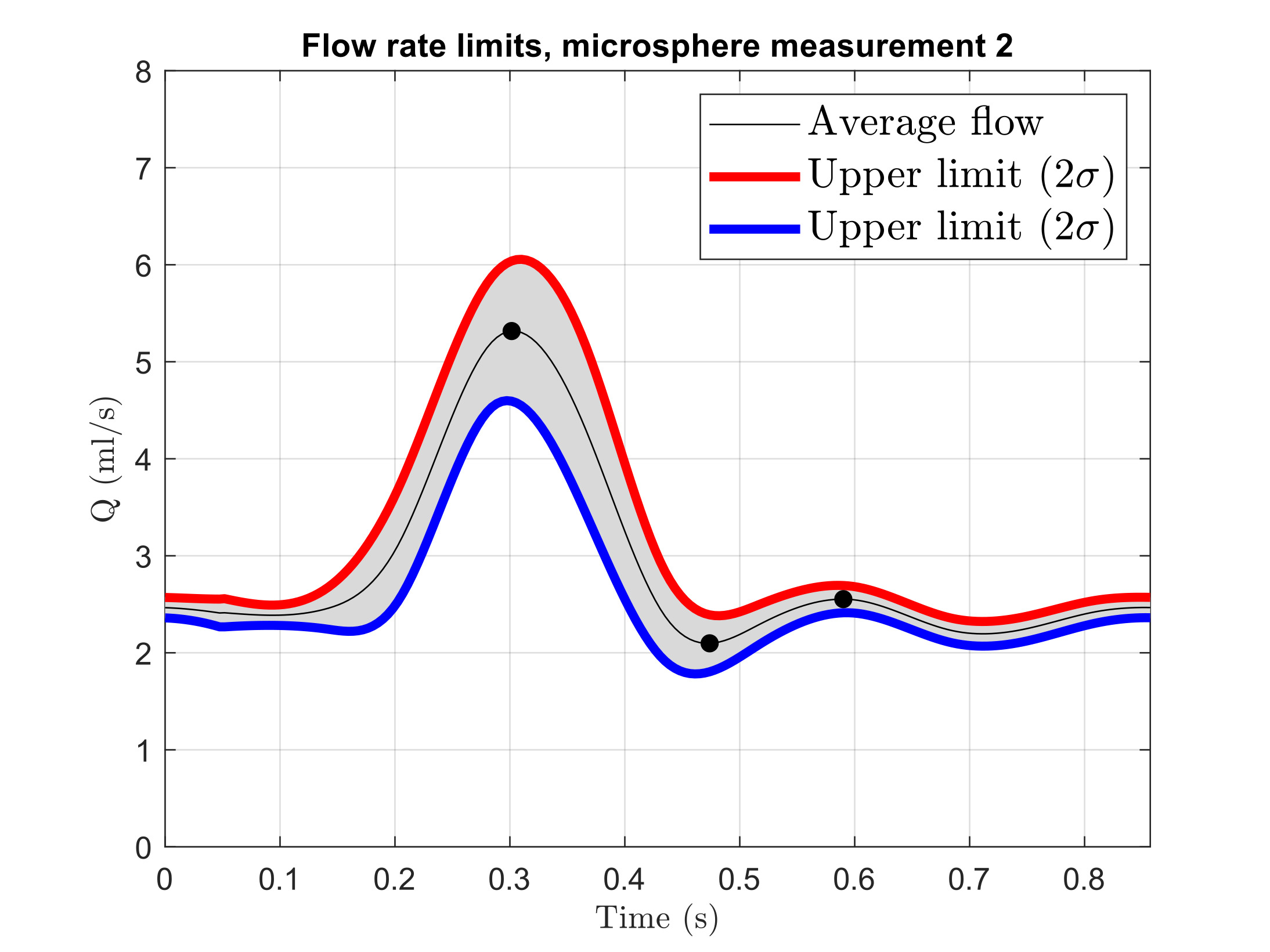

Supplement: Supplemental Material [file IDRD_A_2505007_SM5900.zip › Suppl_Doc/Sup1_Holmium_flow_2.jpg]

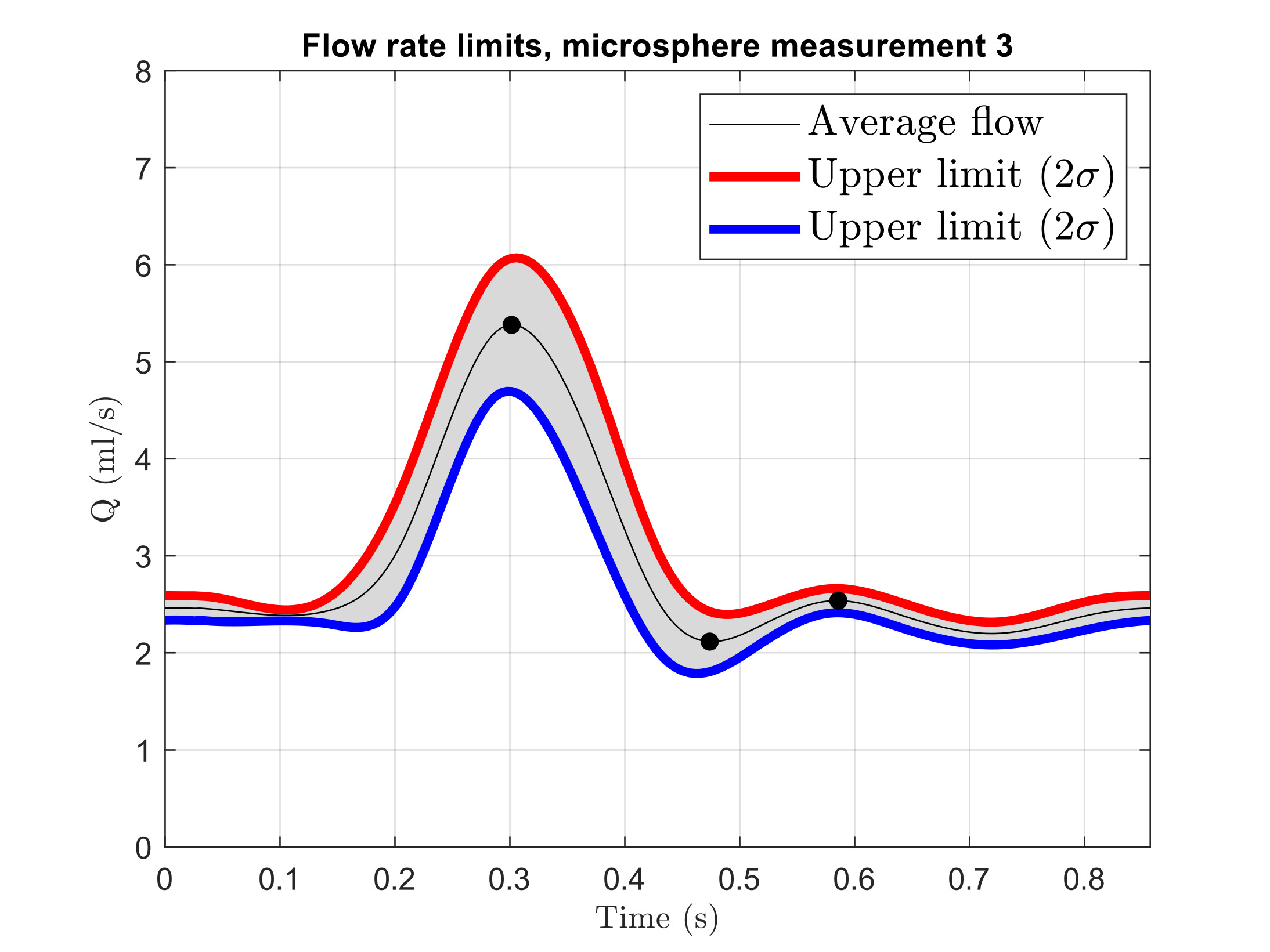

Supplement: Supplemental Material [file IDRD_A_2505007_SM5900.zip › Suppl_Doc/Sup1_Holmium_flow_3.jpg]

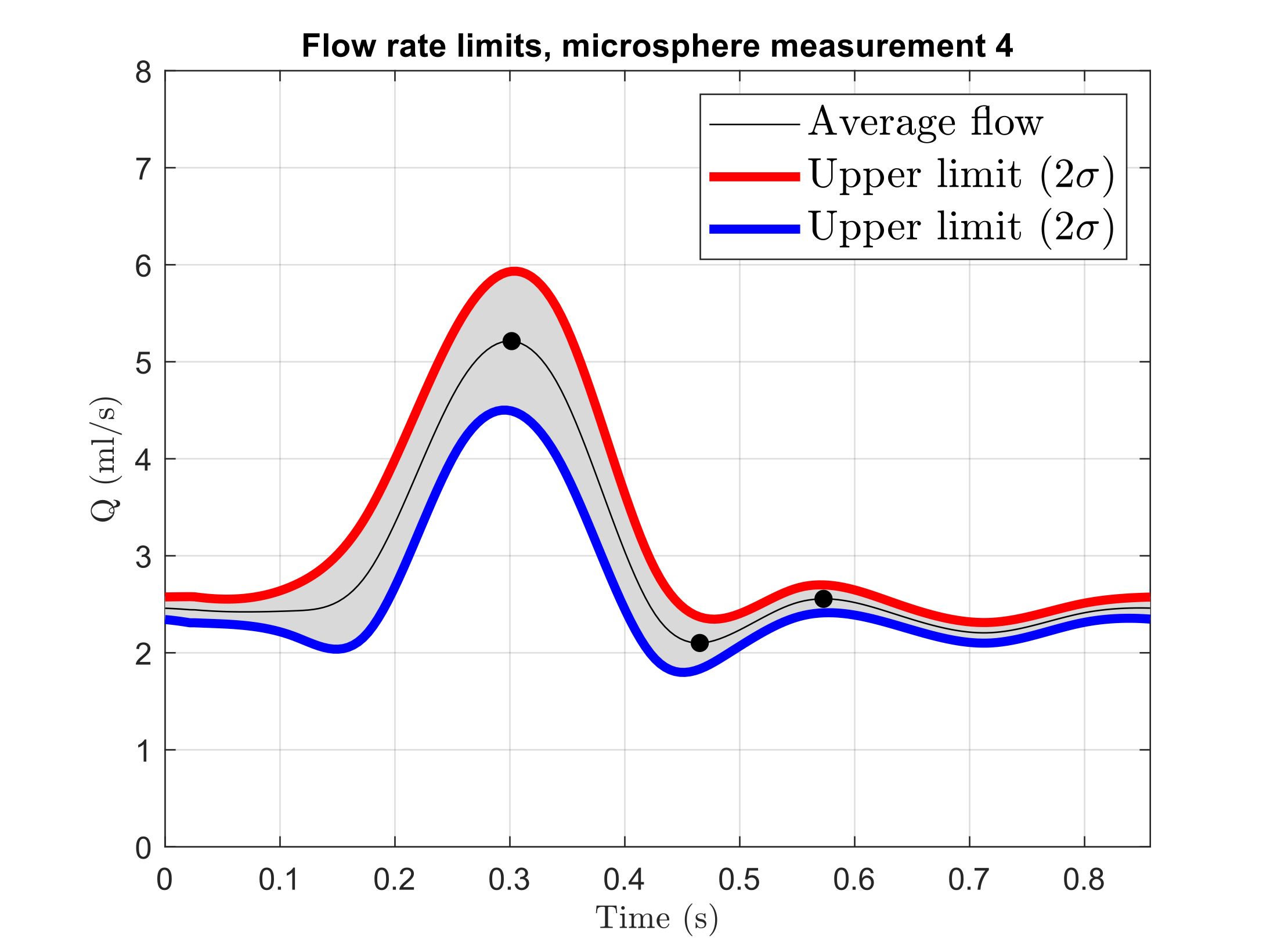

Supplement: Supplemental Material [file IDRD_A_2505007_SM5900.zip › Suppl_Doc/Sup1_Holmium_flow_4.jpg]

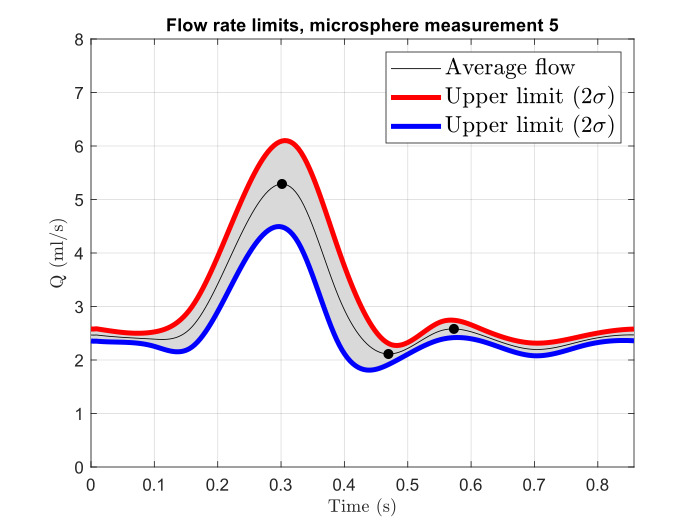

Supplement: Supplemental Material [file IDRD_A_2505007_SM5900.zip › Suppl_Doc/Sup1_Holmium_flow_5.jpg]

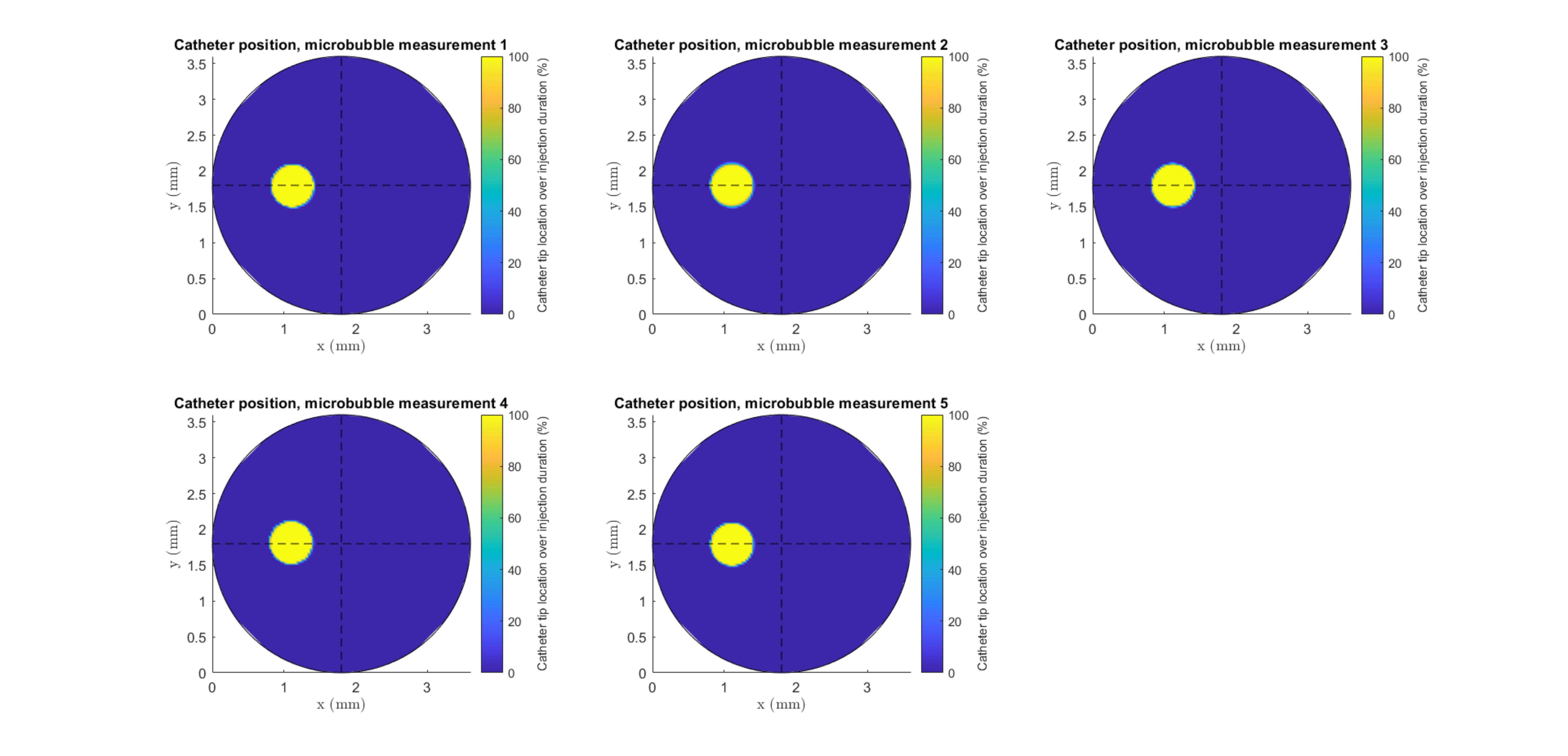

Supplement: Supplemental Material [file IDRD_A_2505007_SM5900.zip › Suppl_Doc/Sup2_Bubble_heatmaps_all.jpg]

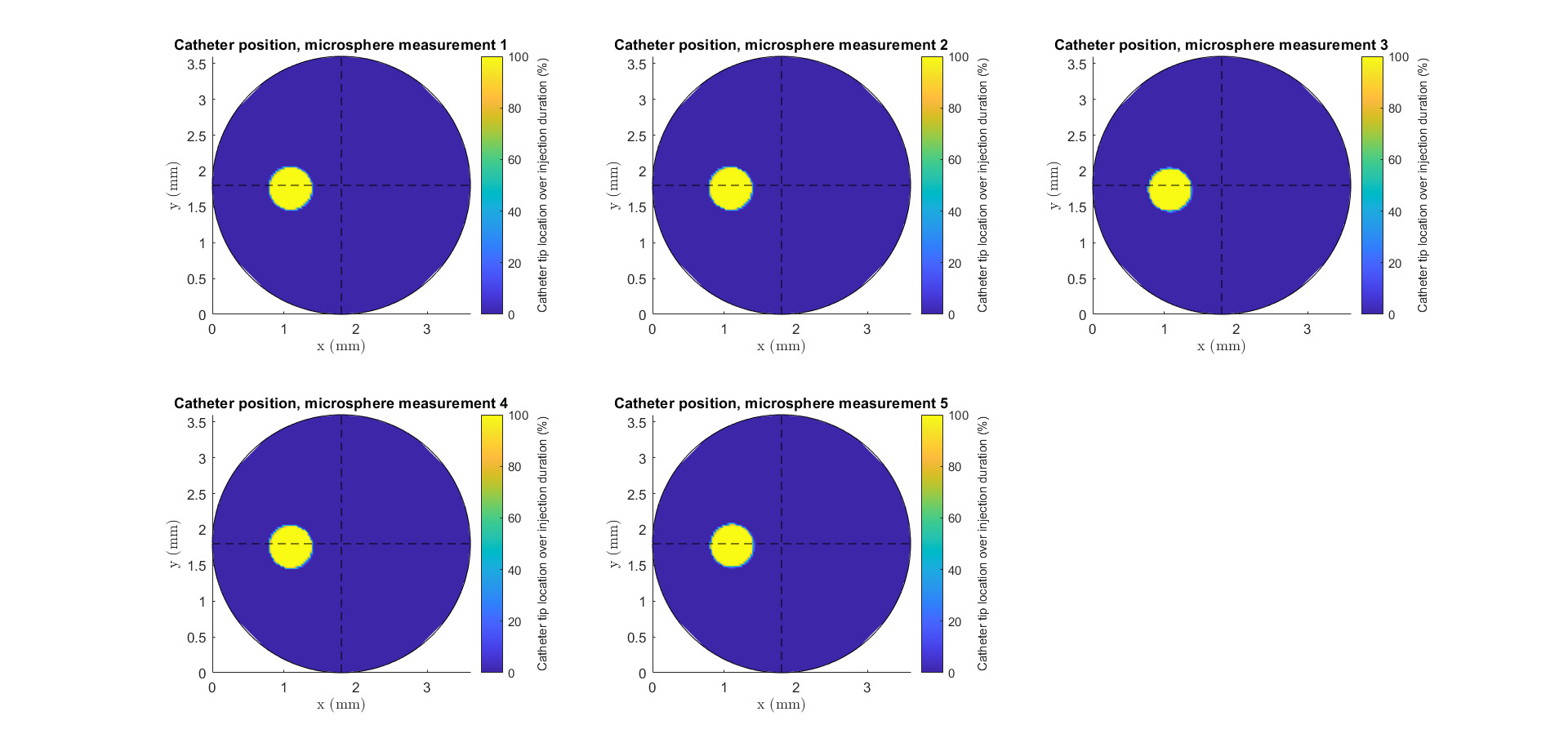

Supplement: Supplemental Material [file IDRD_A_2505007_SM5900.zip › Suppl_Doc/Sup2_Holmium_heatmaps_all.jpg]

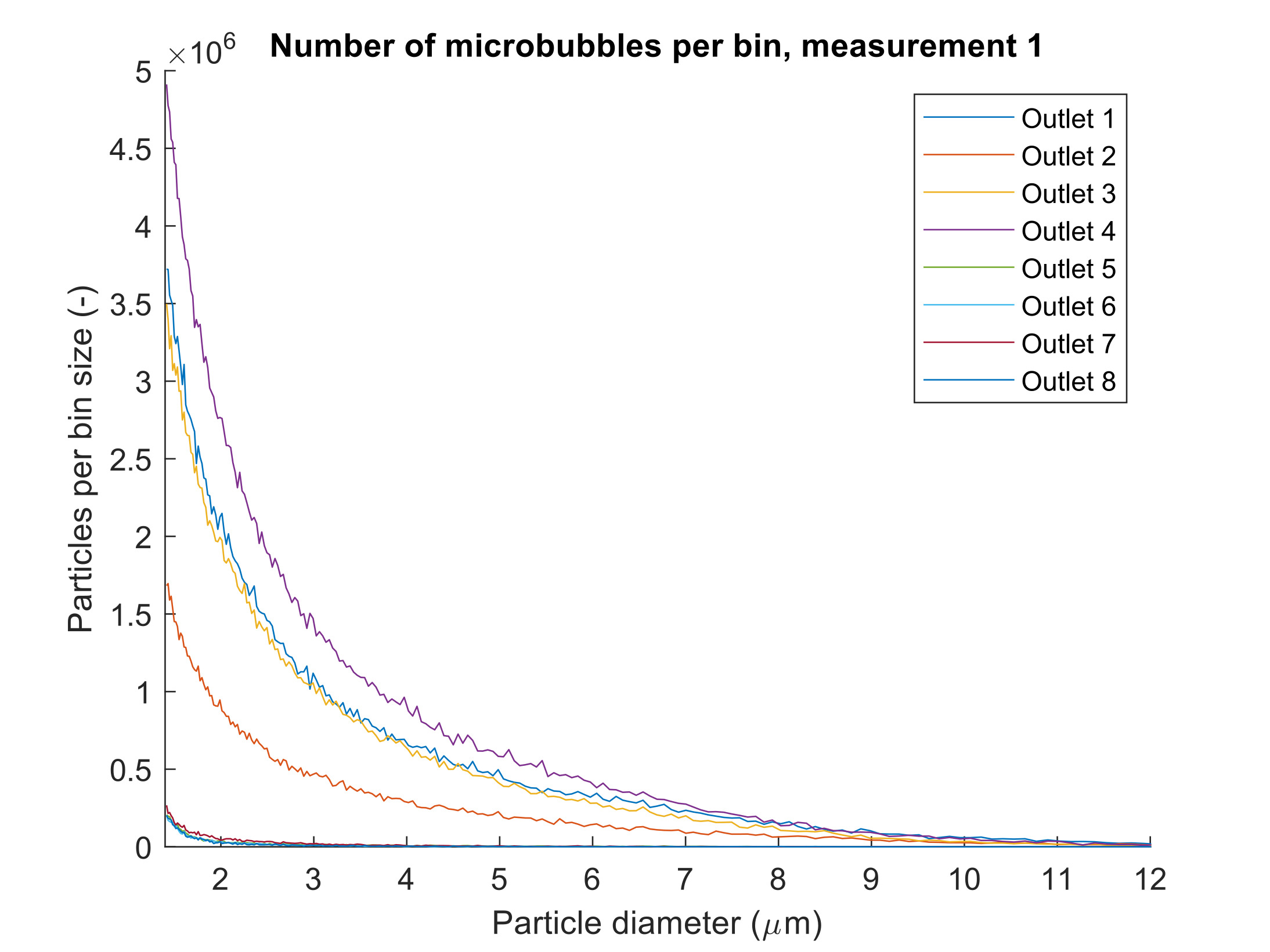

Supplement: Supplemental Material [file IDRD_A_2505007_SM5900.zip › Suppl_Doc/Sup3_Bubble_coulter_1.jpg]

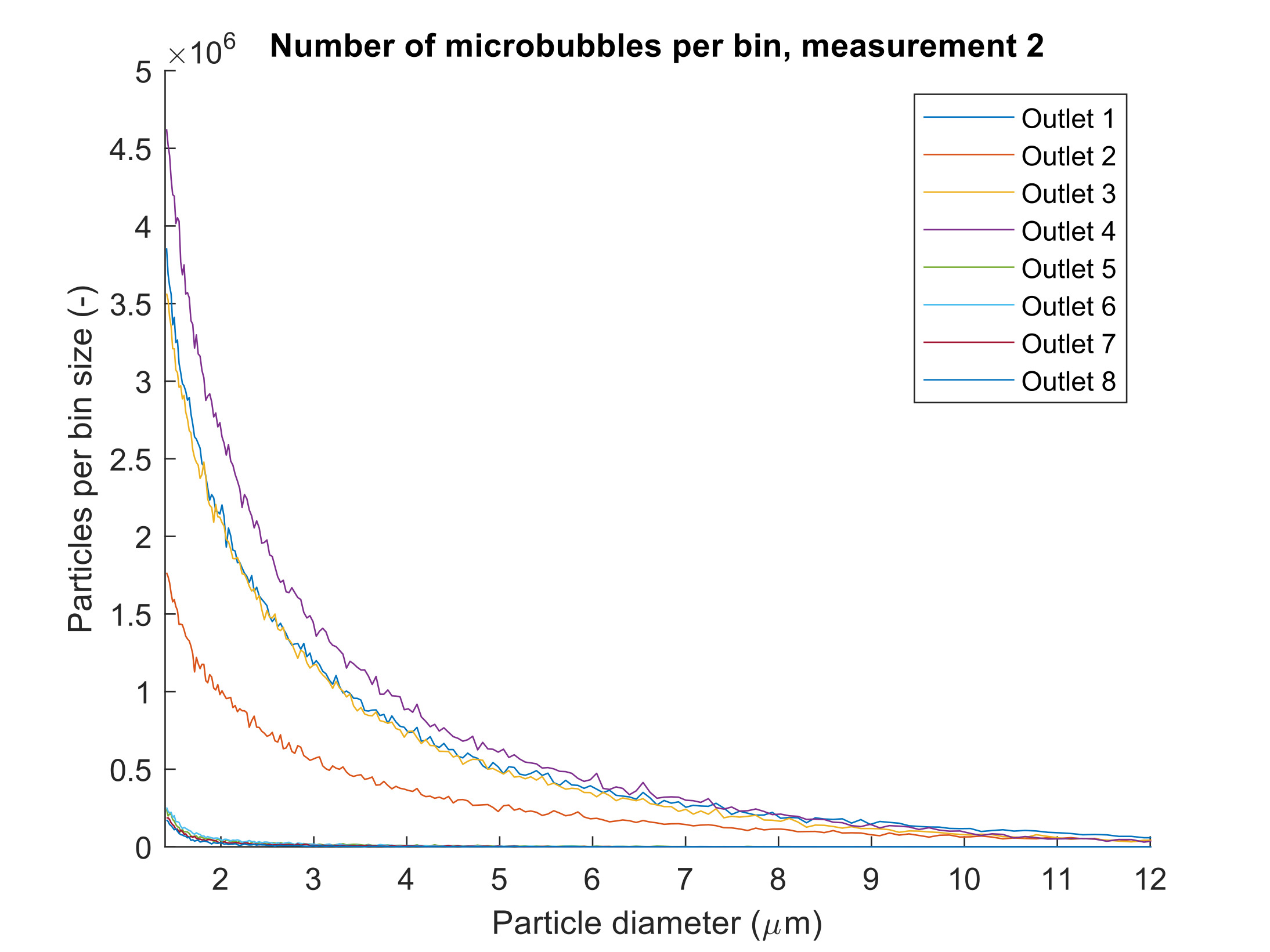

Supplement: Supplemental Material [file IDRD_A_2505007_SM5900.zip › Suppl_Doc/Sup3_Bubble_coulter_2.jpg]

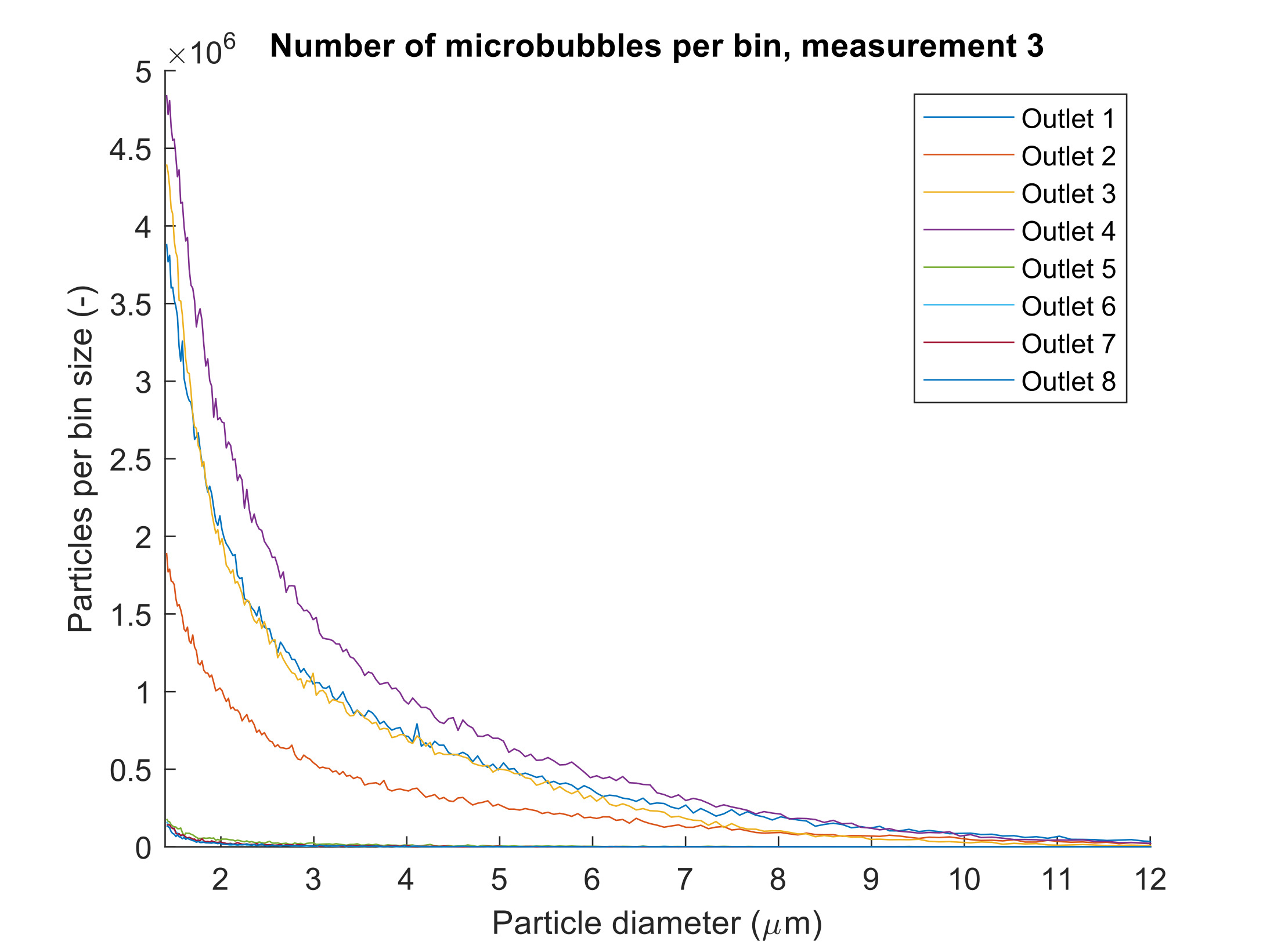

Supplement: Supplemental Material [file IDRD_A_2505007_SM5900.zip › Suppl_Doc/Sup3_Bubble_coulter_3.jpg]

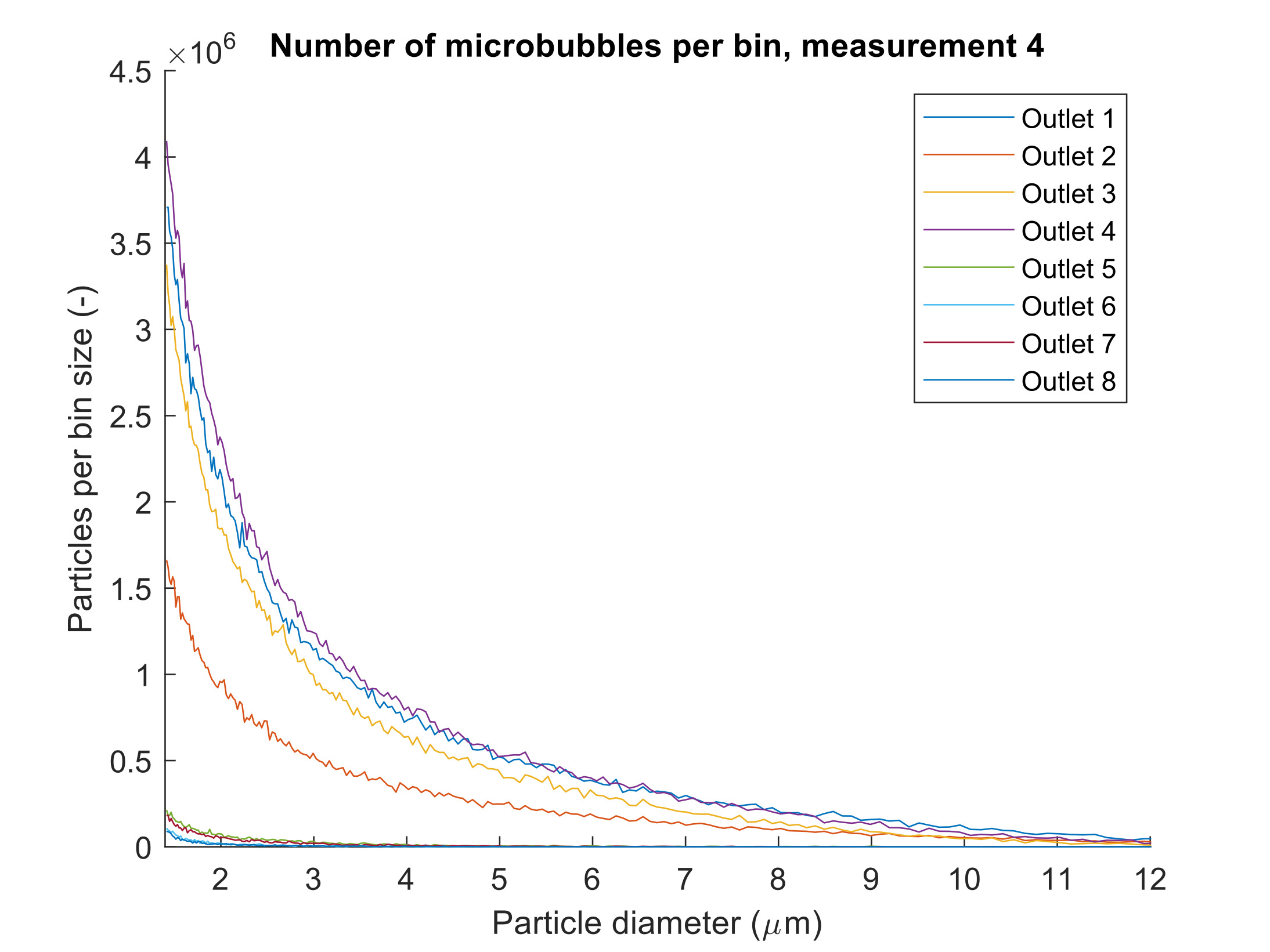

Supplement: Supplemental Material [file IDRD_A_2505007_SM5900.zip › Suppl_Doc/Sup3_Bubble_coulter_4.jpg]

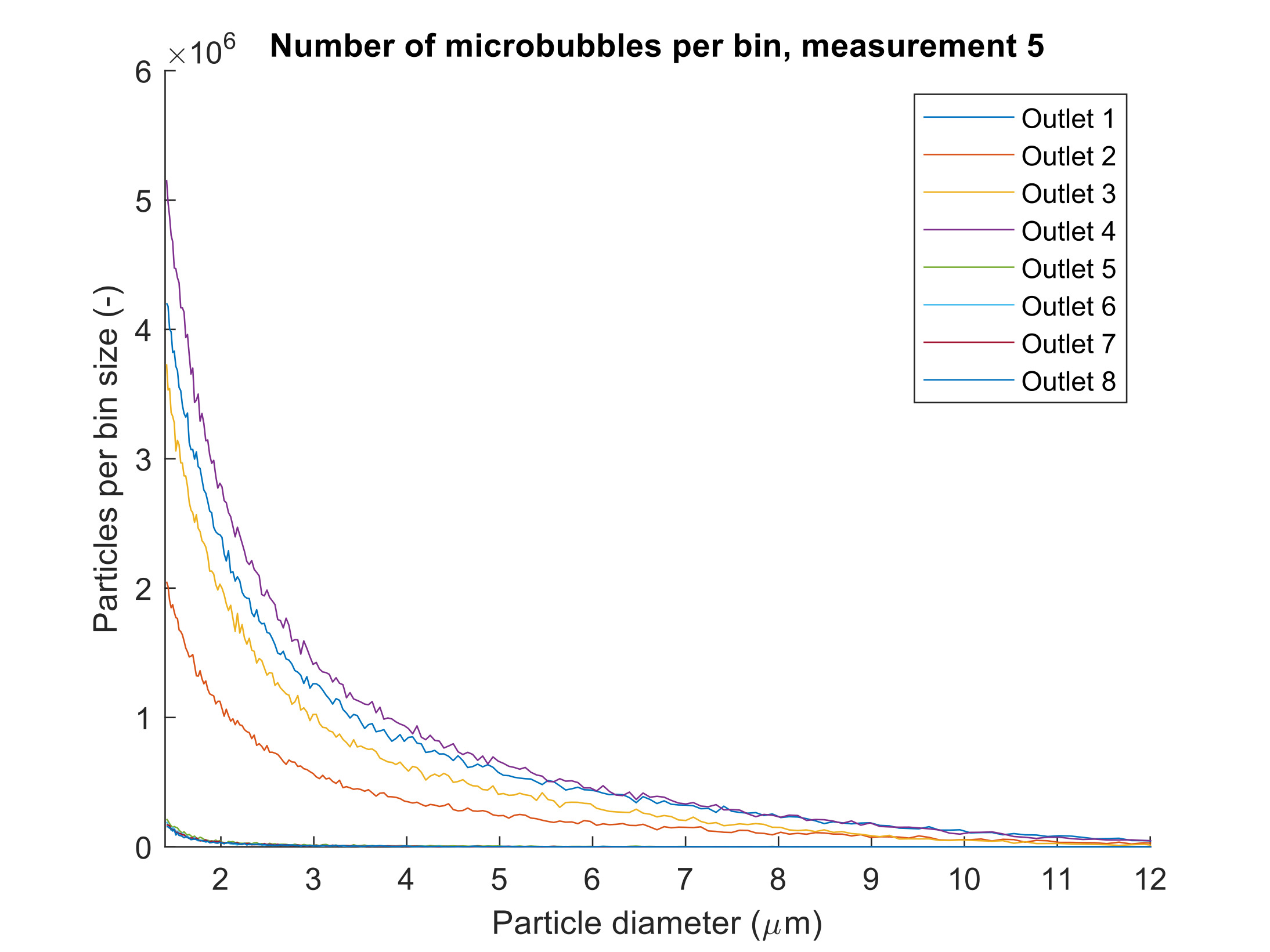

Supplement: Supplemental Material [file IDRD_A_2505007_SM5900.zip › Suppl_Doc/Sup3_Bubble_coulter_5.jpg]

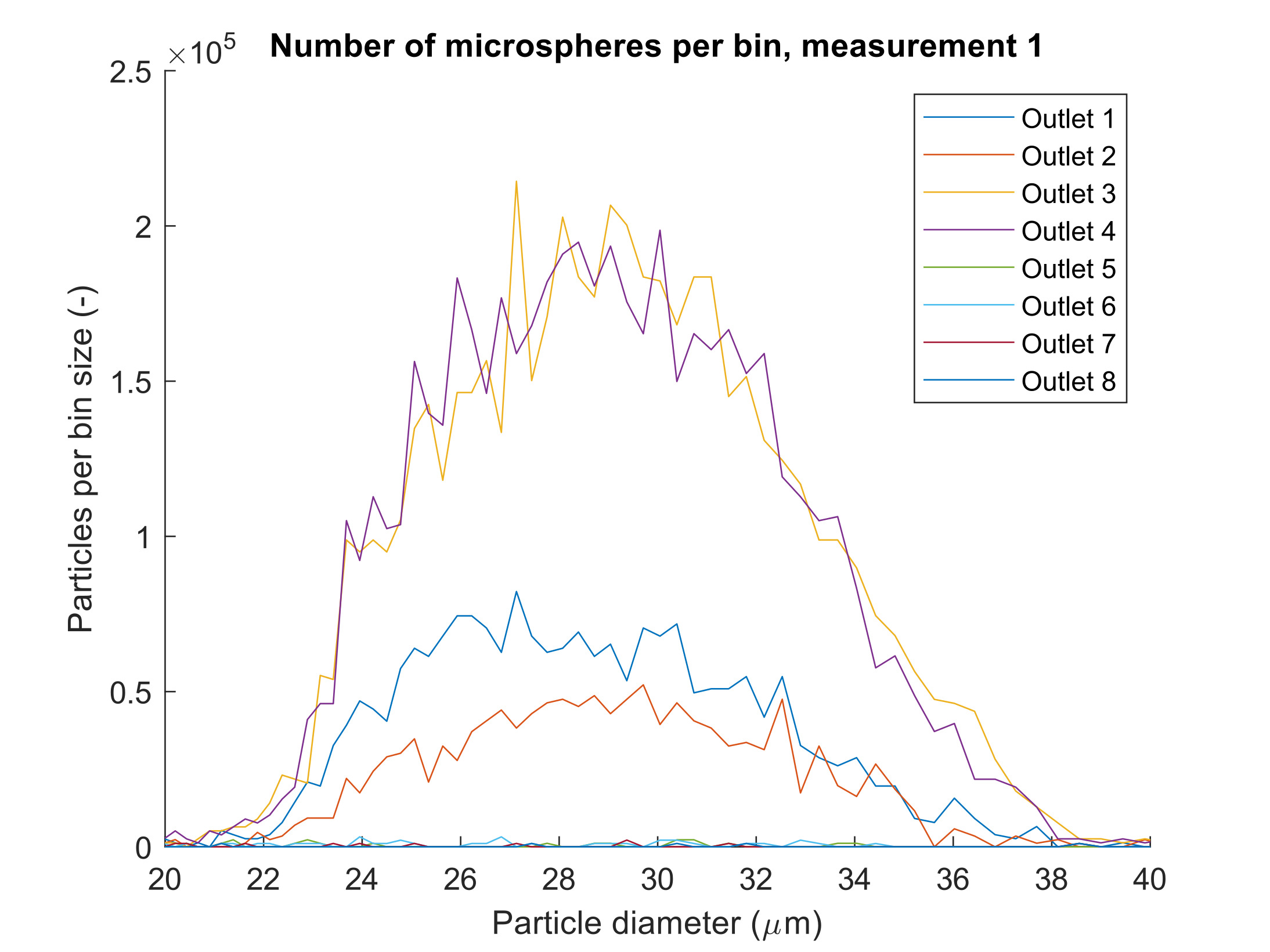

Supplement: Supplemental Material [file IDRD_A_2505007_SM5900.zip › Suppl_Doc/Sup3_Holmium_coulter_1.jpg]

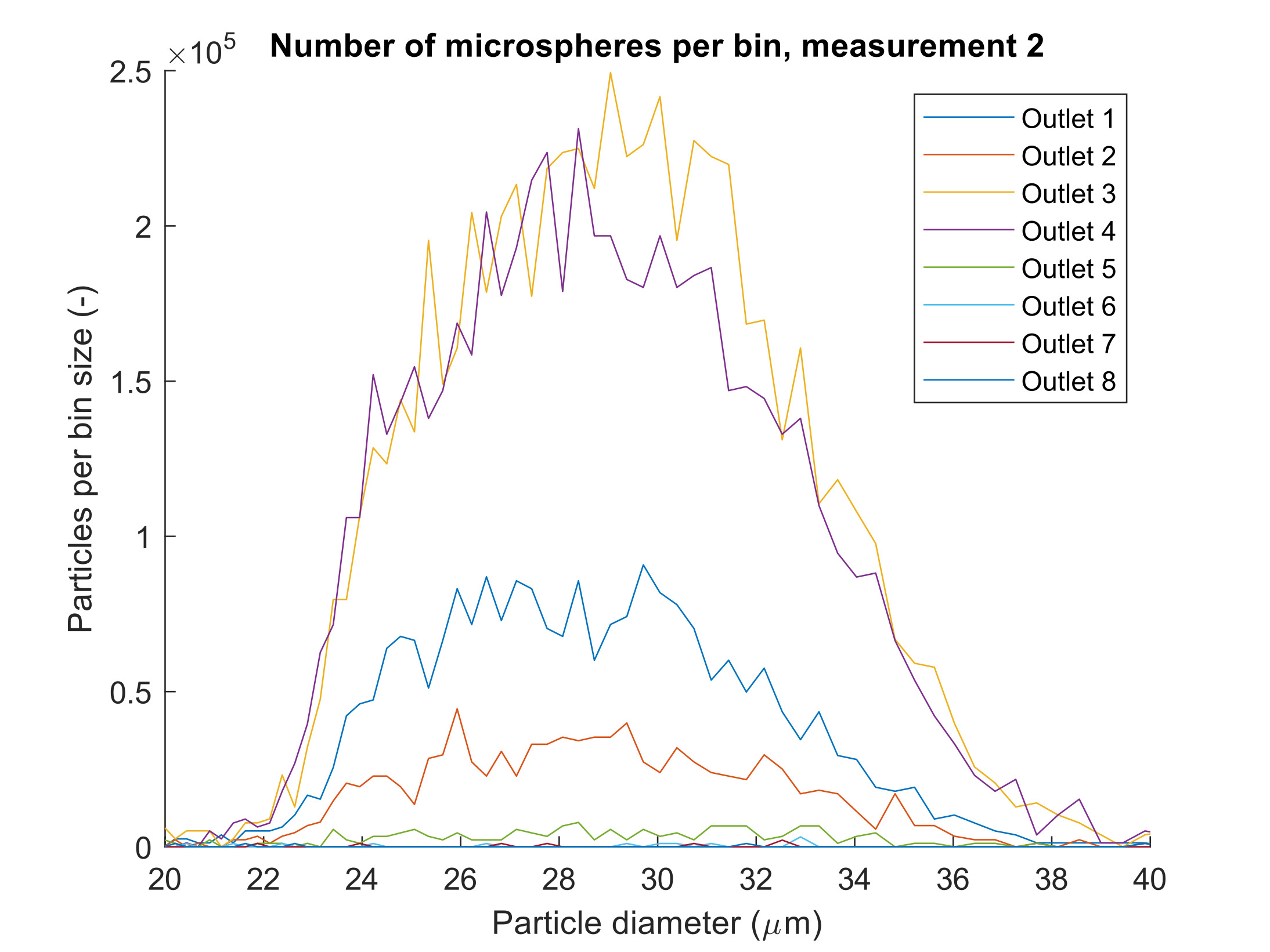

Supplement: Supplemental Material [file IDRD_A_2505007_SM5900.zip › Suppl_Doc/Sup3_Holmium_coulter_2.jpg]

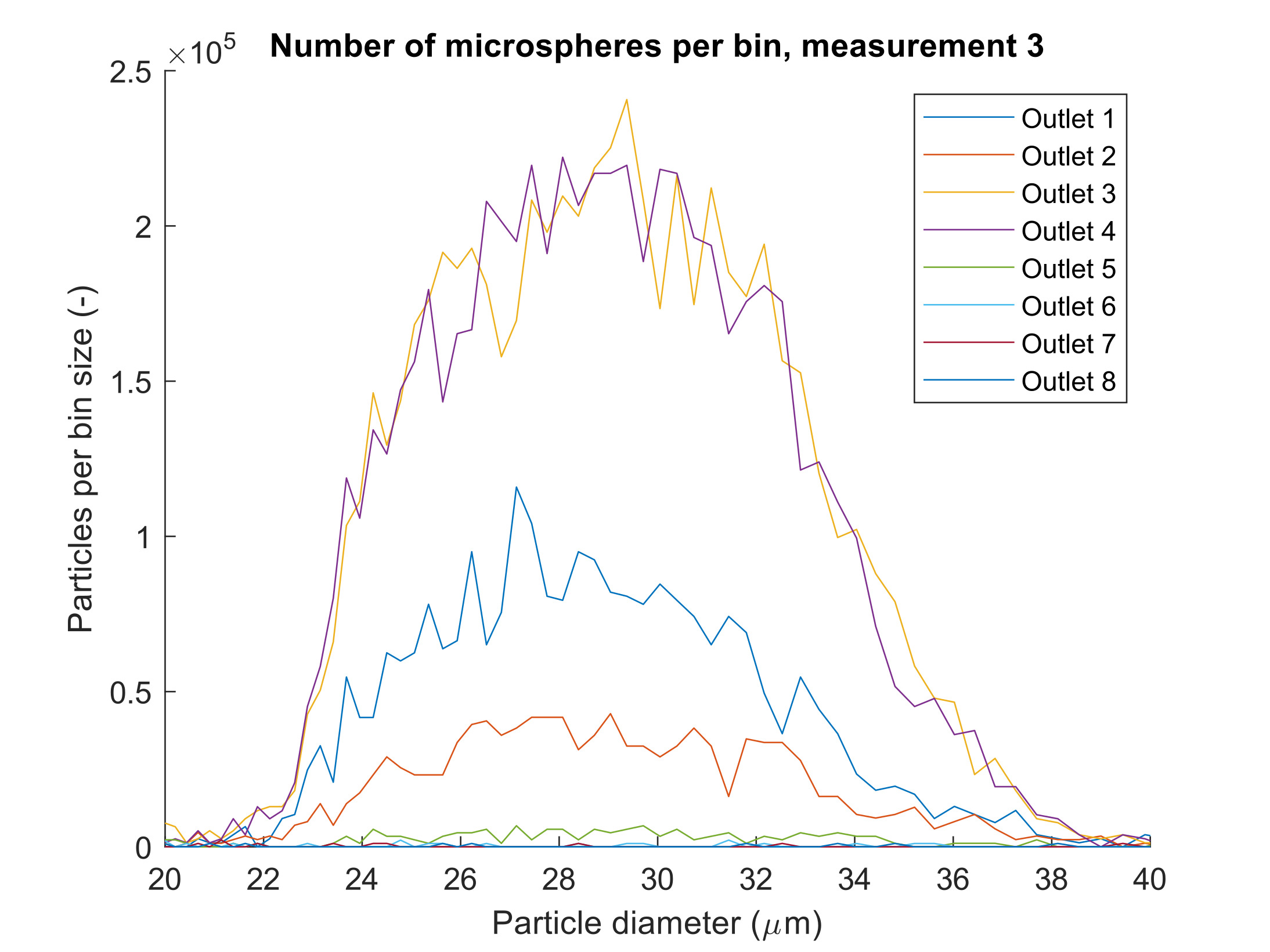

Supplement: Supplemental Material [file IDRD_A_2505007_SM5900.zip › Suppl_Doc/Sup3_Holmium_coulter_3.jpg]

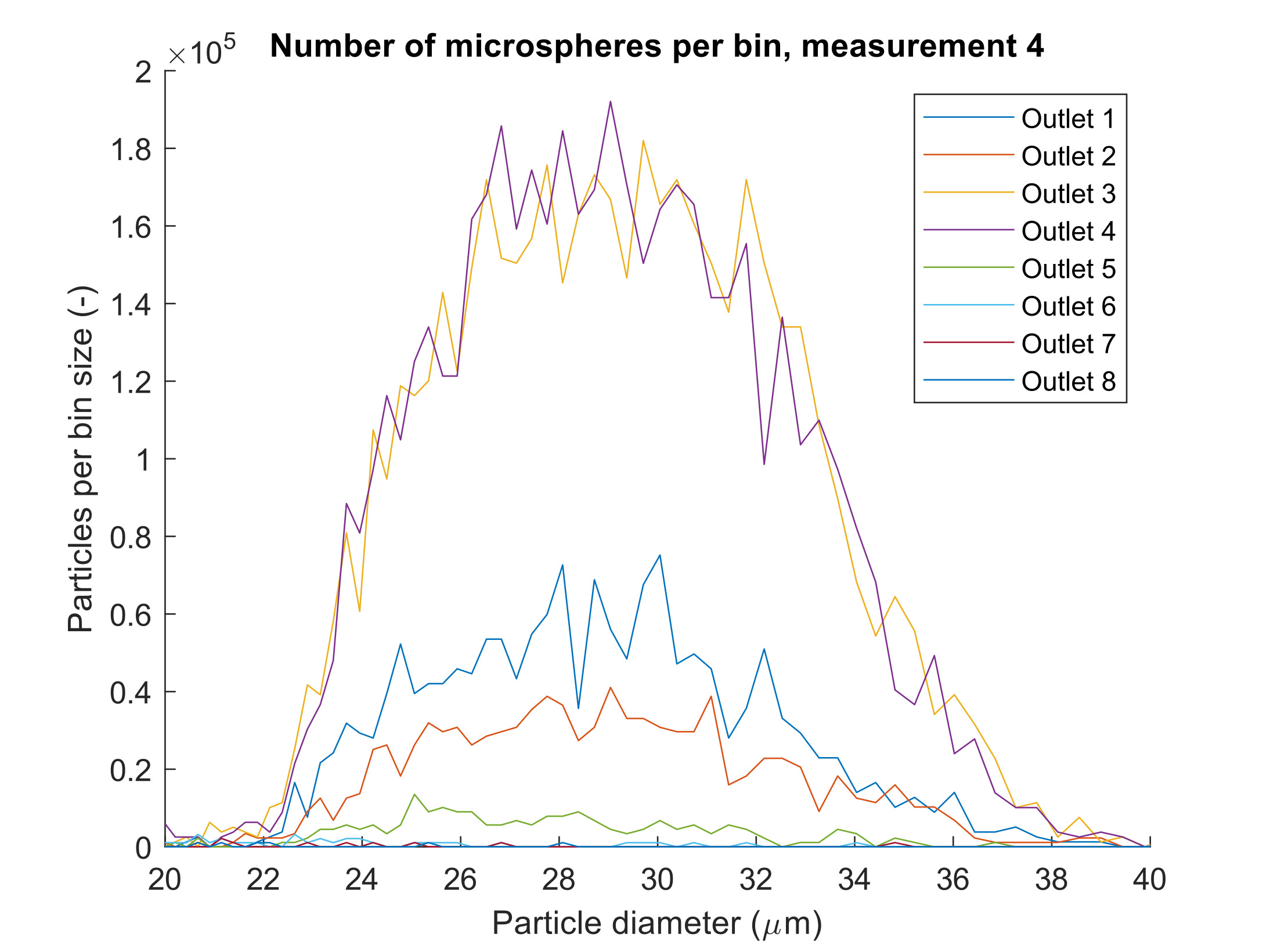

Supplement: Supplemental Material [file IDRD_A_2505007_SM5900.zip › Suppl_Doc/Sup3_Holmium_coulter_4.jpg]

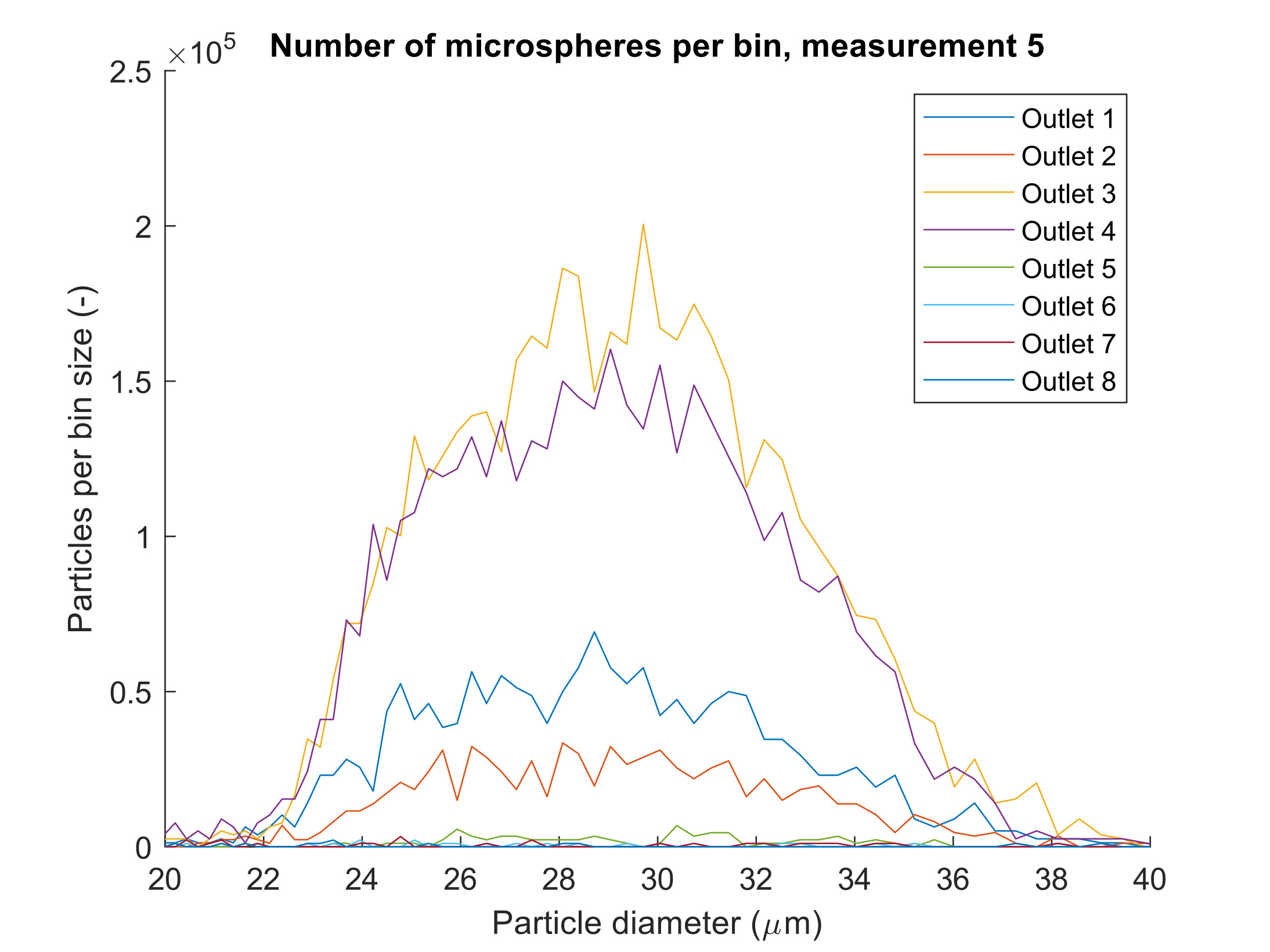

Supplement: Supplemental Material [file IDRD_A_2505007_SM5900.zip › Suppl_Doc/Sup3_Holmium_coulter_5.jpg]
